# Metal–Organic Framework Featuring Monodispersed Silver Cation Sites for Highly Efficient and Selective Extraction of Aqueous Iodide Anions

**DOI:** 10.1002/advs.202517224

**Published:** 2025-10-30

**Authors:** Xuewen Cao, Jiacheng Zhang, Jinjiao Pan, Yan Li, Yue Ma, Xinfeng Du, Lijuan Feng, Boyang Huang, Yihui Yuan, Liang Mao, Ning Wang, Abdullah M. Al‐Enizi, Ayman Nafady, Shengqian Ma

**Affiliations:** ^1^ State Key Laboratory of Marine Resource Utilization in South China Sea Hainan University Haikou 570228 P. R. China; ^2^ State Key Laboratory of Pollution Control and Resource Reuse School of the Environment, Chemistry and Biomedicine Innovation Center Nanjing University Nanjing 210093 P. R. China; ^3^ Department of Chemistry University of North Texas Denton TX 76201 USA; ^4^ Department of Chemistry College of Science King Saud University Riyadh 11451 Saudi Arabia

**Keywords:** iodide ions, metal‐organic frameworks, monodispersed function sites, seawater, site‐specific

## Abstract

Efficient extraction of iodide ion (I^−^), the dominant form of aquatic iodine, is crucial for both resource recovery and pollution mitigation, particularly in seawater, nuclear wastewater, and drinking water. However, this remains a significant challenge due to the lack of adsorbents that simultaneously offer high affinity, fast kinetics, and reliable reusability for I^−^ capture. Herein, a novel cationic metal–organic framework featuring monodispersed silver (Ag^+^) sites (MOF‐monoAg), which outperforms conventional Ag‐loaded materials that rely on post‐synthetic loading, enabling uniformly dispersed and highly accessible binding sites for selective and efficient I^−^ extraction, is presented. The unique Ag^+^ sites exhibit extraordinary binding affinity and utilization efficiency, achieving an exceptional distribution coefficient (*K_d_*) of 1.16 × 10^4^ mL g^−1^ and ultrafast adsorption rate of 10.48 mg g^−1^ min^−1^. MOF‐monoAg shows a breakthrough iodine extraction capacity of 114.2 mg g^−1^ in natural seawater, over 50 times the iodine concentration in seaweed, and a 98.7% removal rate for I^−^ in simulated nuclear wastewater. Additionally, MOF‐monoAg‐based membrane effectively reduces the I^−^ concentrations in iodine‐exposed water to below drinking water standards, providing a promising solution for iodine resource recovery and iodine pollution remediation.

## Graphical abstract

A novel cationic metal–organic framework (MOF‐monoAg) featuring monodispersed silver (Ag^+^) sites is developed for the highly selective extraction of aqueous iodide ions (I^−^). MOF‐monoAg exhibits high accessibility, selectivity, and binding affinity toward I^−^ ions, enabling superior iodine extraction from seawater and efficient iodide removal from iodide‐contaminated water, significantly surpassing conventional adsorbents.

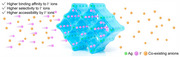

## 1 Introduction

Iodine is an indispensable strategic element with diverse industrial, medical, and nutritional applications.^[^ ^1^, ^2^, ^3^ ^]^ The global demand for iodine is projected to grow at a compound annual growth rate (CAGR) of 5.41% over the next five years.^[^ ^4^ ^]^ However, iodine is among the less abundant nonmetallic elements on Earth.^[^ ^5^ ^]^ The currently available approaches for accessing iodine resources mainly depend on the cultivation of seaweed and the mining of iodine from iodine‐rich minerals and underground brines.^[^ ^6^, ^7^, ^8^ ^]^ Seawater, the largest global iodine reservoir, is estimated to contain ≈90 billion tons of iodine, offering enormous potential for iodine resource development.^[^ ^9^ ^]^ Furthermore, radioactive iodine isotopes, such as I‐129 and I‐131, are high‐yield fission products generated from uranium during nuclear energy production.^[^ ^10^ ^]^ The occurrence of nuclear accidents, such as the Fukushima nuclear accident, has led to the leakage of radioactive iodine into seawater, exacerbating environmental and health concerns. During the past few decades, the concentration of radioactive iodine in natural seawater has increased by one order of magnitude.^[^ ^11^ ^]^ Given iodine's essential roles in various biological processes, including the life activities of seaweed and the function of participating in the synthesis of thyroid hormone, the ingestion of radioactive iodine would cause significant health risks to biological entities.^[^ ^12^, ^13^, ^14^ ^]^ In addition, iodine is widely used for drinking water disinfection. However, due to the lack of effective technologies for removing low concentrations of iodine, the residual iodine in drinking water has become a significant health risk. Therefore, developing efficient iodine recovery methods is of great significance for the sustainable development of iodine resources, remediation of nuclear iodine pollution, and treatment of iodine contamination in drinking water.

**Figure 1:**
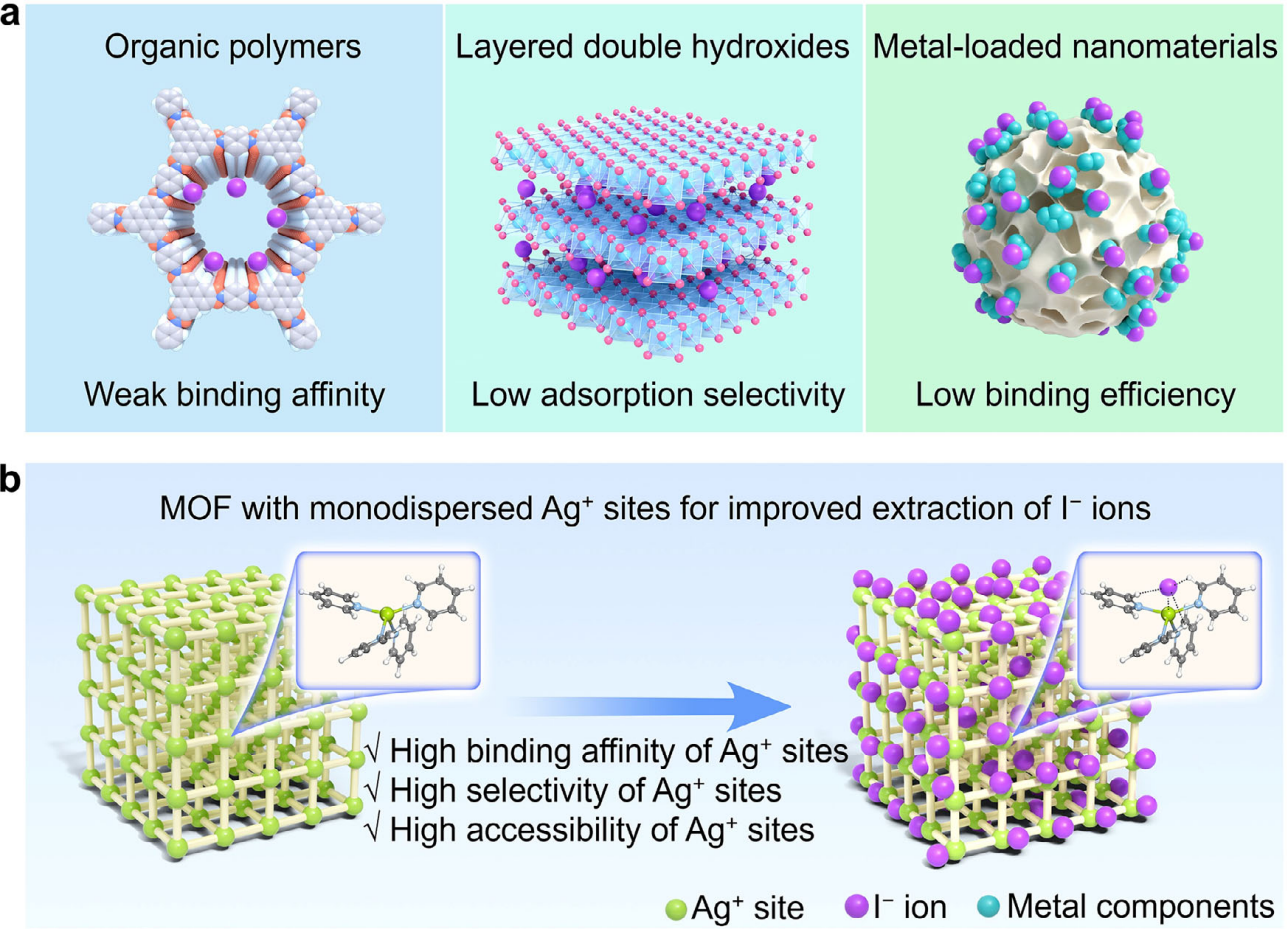
Comparison of the features of previously reported adsorbents and the adsorbent designed in this study for I^−^ ions recovery. a) Shortages of organic polymers, layered double hydroxides, and metal‐loaded nanomaterials for recovery of I^−^ ions (Color code: C, gray; H, white; O, red; N, blue; OH^−^, pink; M^2+^/M^3+^, light blue; Metal components, cyan). b) Schematic diagram for the improved extraction performance for I^−^ ions of the MOF adsorbent designed in this study with monodispersed functional Ag^+^ sites.

In natural environments, iodine primarily exists as iodine (I_2_), iodide ion (I^−^), iodate ion (IO_3_^−^), and hypoiodite ion (IO^−^).^[^ ^15^, ^16^ ^]^ I^−^ and IO_3_^−^ ions are the predominant iodine species in aqueous environments, each accounting for ≈50% of the total iodine.^[^ ^16^, ^17^, ^18^ ^]^ These two iodine species exist in chemical equilibrium and can transform from one species to another along with the change of environmental conditions.^[^ ^17^ ^]^ Thus, the utilization of seawater iodine resources can focus on the recovery of either I^−^ or IO_3_^−^ ions. However, there are rare reports on the recovery of IO_3_^−^ ions from natural aqueous environments, primarily due to the complex structure of IO_3_^−^ ion and the absence of specific recognition functional groups.^[^ ^19^ ^]^ Furthermore, the recovery of aqueous I^−^ ions is also technically challenging, as the spherical charge distribution of I^−^ ion complicates the design of functional recognition groups for selective binding to I^−^ ion.^[^ ^20^ ^]^ To date, various materials, including organic polymers, layered double hydroxides (LDHs), and metal‐loaded nanomaterials, have been explored for the adsorption of aqueous I^−^ ions (**Figure** **1**a). Among them, organic polymers adsorb I^−^ ions primarily through hydrogen bonding, where the lone pair electrons of I^−^ ion act as a hydrogen bond receptor to bind with the hydrogen atoms in the organic polymers.^[^ ^21^, ^22^, ^23^ ^]^ However, due to the low electronegativity of I^−^ ion, the binding affinity and selectivity of organic polymers for I^−^ ion remain insufficient, limiting their applicability in complex real‐world environments. LDHs materials adsorb I^−^ ions via anion exchange, while the low charge density of I^−^ ion reduces its competitiveness against higher charge density ions, and its relatively large ionic radius restricts the entrance into the interlayer spaces of LDHs materials, which leads to low adsorption selectivity and capacity.^[^ ^24^, ^25^ ^]^ The adsorption ability of metal‐loaded nanomaterials for I^−^ ions relies on the formation of stable complexes between the loaded metal components and I^−^ ions.^[^ ^26^, ^27^, ^28^ ^]^ However, these metal elements (including Ag, Cu, and Bi) are typically loaded through post‐synthetic chemical deposition and tend to aggregate on the supporting materials, reducing the utilization efficiency of the metal components. Moreover, the resulting products irreversibly occupy the active sites, making the materials difficult to regenerate or reuse. Consequently, the application of specific I^−^ ion‐recognizing functional groups coupled with the rational design of adsorbent structures to improve I^−^ ion binding affinity, selectivity, reusability, as well as the utilization efficiency of the functional groups would be a promising strategy to realize highly efficient I^−^ ion recovery.

Attributing to the feature as soft acid, the silver ion (Ag^+^) is recognized as an efficient functional site for binding soft base I^−^ ion, and various adsorbents using Ag^+^ ions as functional sites have been designed. However, in the currently available adsorbents, Ag^+^ sites are randomly loaded through depositions, and Ag^+^ sites tend to aggregate in the adsorbents, leading to the low utilization efficiency of the functional sites. Such aggregation also causes partial blockage of micropores, which diminishes the accessibility of the pores and undermines the fast mass transfer required for efficient adsorption. Thus, these available adsorbents based on Ag^+^ sites only show limited adsorption efficiency and a slow adsorption rate.^[^ ^29^, ^30^, ^31^ ^]^ Cationic metal–organic frameworks (MOFs) are highly potential alternatives for application in recovering environmental anions due to their structure controllability and space availability.^[^ ^32^, ^33^, ^34^ ^]^ The well‐defined spatial architecture of MOFs can enable the uniform anchoring of Ag^+^ sites, preventing their aggregation and ensuring high utilization efficiency of these functional sites. The positively charged metal nodes serve as the functional sites for binding negatively charged I^−^ ions, while the customizable spatial structure provides channels and cavities for the ingress and entrapment of I^−^ ions within the material.

In this work, we synthesized a cationic MOF, denoted as MOF‐monoAg, by using Ag^+^ ions as connection nodes to facilitate efficient adsorption of I^−^ ions (Figure 1b). MOF‐monoAg incorporates monodispersed Ag^+^ sites within a well‐defined framework, addressing the limitations of conventional Ag‐loaded adsorbents by combining high selectivity, rapid kinetics, and high regenerability for efficient I^−^ capture. MOF‐monoAg exhibits the highest reported distribution coefficient (*K*_d_) of 1.16 x 10^4^ mL g^−1^ and the fastest adsorption rate of 10.48 mg g^−1^ min^−1^ for aqueous I^−^ ions. Notably, it shows excellent potential for practical applications across diverse aqueous environments. A high removal efficiency of 98.7% for I^−^ ions in simulated Hanford wastewater is achieved. In natural seawater, MOF‐monoAg exhibits an outstanding iodine extraction capacity of 114.2 mg g^−1^ within four days, which is 50 times higher than the iodine content in seaweed. Furthermore, we developed a composite MOF‐monoAg@PVDF membrane to construct a dynamic membrane filtration system (DMFS) for the decontamination of iodide‐exposed drinking water. The DMFS, based on the composite membrane, demonstrates high rejection efficiency and selectivity for I^−^ ions, reducing its concentration to levels below the Canadian screening threshold established for drinking water. The findings of this study will stimulate future research into addressing iodine pollution and ensuring the sustainable supply of iodine resources.

## 2 Results and Discussion

### 2.1 Synthesis and Characterization of MOF‐monoAg

**Figure 2:**
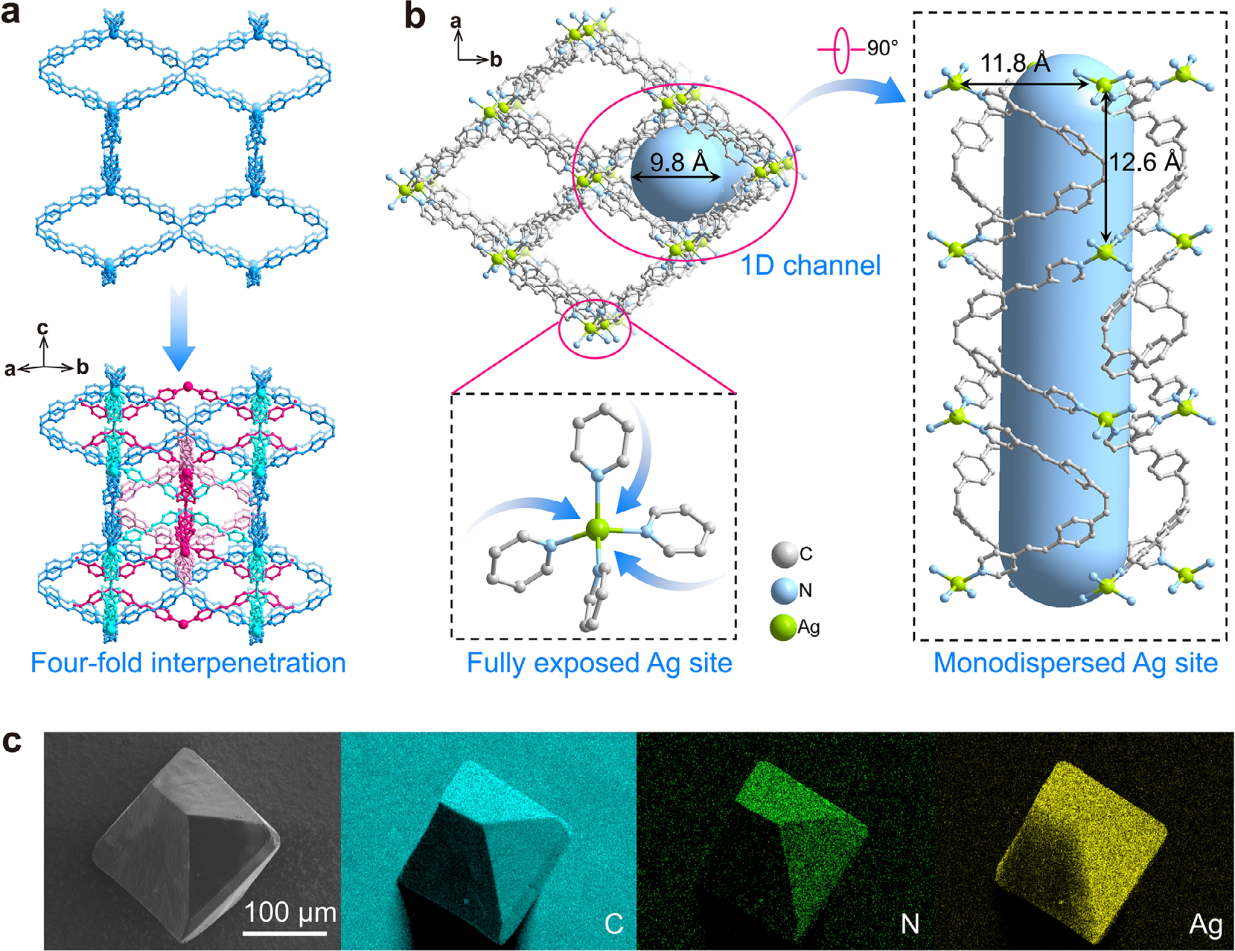
Structure and properties of MOF‐monoAg. a) Four fold interpenetration topology of MOF‐monoAg viewed along the [110] direction. b) 1D channels and fully exposed monodispersed Ag^+^ sites in MOF‐monoAg viewed along the [001] direction. c) Morphology and corresponding EDS mapping of MOF‐monoAg.

MOF‐monoAg was synthesized by using organic ligand 1,1,2,2‐Tetrakis(4‐((E)‐2‐(pyridin‐4‐yl)vinyl)phenyl)ethene (TPVPE) and metal node donor AgBF_4_ through a solvothermal reaction (Figure S1, Supporting Information). The as‐synthesized MOF‐monoAg exists as a yellow octahedron crystal and features a porous 3D skeleton with the pts topology structure (**Figure** **2**a; Table S1, Supporting Information). Powder X‐ray diffraction (PXRD) analysis of MOF‐monoAg is consistent with the simulated data obtained from the single crystal X‐ray diffraction analysis, revealing that the prepared material exists in the pure phase (Figure S2, Supporting Information). As for the spatial structure of MOF‐monoAg, each Ag^+^ ion coordinates with four nitrogen atoms from the organic ligand TPVPE via the Ag─N coordination bonds with lengths of 2.32 and 2.35 Å. The monolayer network of MOF‐monoAg possesses a large pore size of 36.8 Å along the [110] direction and further interpenetrates to form a four fold 3D structure (Figure 2a; Figure S3, Supporting Information). Such an interpenetration arrangement leads to the 1D channel of 9.8 Å along the [001] direction, which is sufficient for the diffusion of I^−^ ions with an ionic radius of ≈2.2 Å within the framework (Figure 2b; Figure S4, Supporting Information). The four fold interpenetration of the monolayer networks endows the adjacent Ag nodes with distances of 11.8 and 12.6 Å, forming monodispersed Ag^+^ sites that provide sufficient space for the full binding of I^−^ ion to each Ag^+^ site. Scanning electron microscopy (SEM) and energy‐dispersive X‐ray spectroscopy (EDS) analysis also prove the octahedral morphology and the uniform distribution of silver element in MOF‐monoAg (Figure 2c). Electrostatic potential distribution (ESP) mapping of the optimized geometrical configuration of the partial framework shows that the Ag^+^ site provides the highest positive ionization energy, which is desirable for binding the negative I^−^ ion (Figures S5 and S6, Supporting Information). Fourier‐transform infrared spectroscopy (FTIR) analysis reveals that the peak of the C═N stretching vibration shifts from 1591 cm^−1^ in the spectrum of TPVPE to 1600 cm^−1^ in the spectrum of MOF‐monoAg, which is due to the coordination between the Ag^+^ ion and the pyridine N in TPVPE. Additionally, a new peak at 1054 cm^−1^ is observed, corresponding to the BF_4_^−^ ion, which confirms the presence of the counterions within the framework (Figure S7, Supporting Information). The Brunauer−Emmett−Teller (BET) surface area is calculated from the N_2_ adsorption isotherm to be 46.2 m^2^ g^−1^ and the total pore volume is determined to be 0.15 cm^3^ g^−1^ (Figure S8, Supporting Information). The pore size distribution shows a dominant aperture of 9.8 Å, which matches well with the 1D open channels observed along the *c* axis of MOF‐monoAg (Figure S9, Supporting Information). Thermogravimetric analysis shows a high thermal stability with the decomposition temperature being as high as 375 °C, thereby confirming the good thermal stability of MOF‐monoAg (Figure S10, Supporting Information). To evaluate the stability of MOF‐monoAg under various aqueous environments, its PXRD patterns, FTIR spectra, and Ag^+^ leaching behavior were systematically examined after immersion in different solutions for 12 h. MOF‐monoAg shows no discernible changes in its PXRD patterns and FTIR spectra, combined with negligible Ag^+^ leaching across pH 7–11 as well as in simulated nuclear wastewater and seawater, demonstrating the high application potential of MOF‐monoAg under tested conditions (Figures S11–S13, Supporting Information).

### 2.2 Adsorption Performance of MOF‐monoAg for Aqueous Iodide

**Figure 3:**
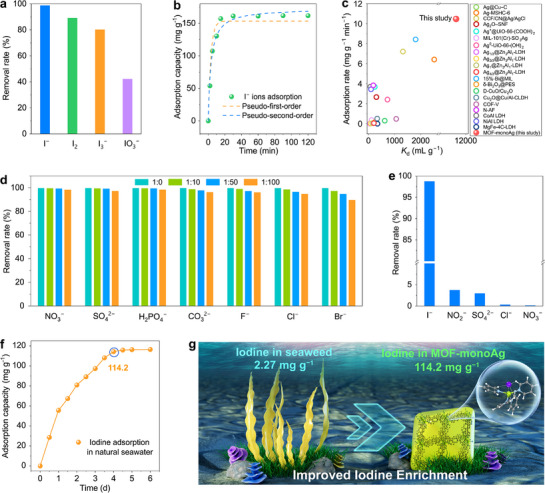
Adsorption performance of MOF‐monoAg for I^−^ ions. a) Removal ability of MOF‐monoAg for four iodine species in aqueous solution. b) Adsorption kinetics of MOF‐monoAg for I^−^ ions in aqueous solution. c) Comparison of *K*_d_ values and adsorption rates for I^−^ ions in aqueous solution of available adsorbents. d) Anti‐interference ability of MOF‐monoAg for I^−^ ions against other co‐existing anions. e) Removal rates of MOF‐monoAg for I^−^ ions and various co‐existing anions in simulated nuclear wastewater. f) Adsorption kinetics of MOF‐monoAg for iodine from natural seawater. g) Comparison of the iodine immobilization capacity of MOF‐monoAg with seaweed in natural seawater.

The iodine removal ability of MOF‐monoAg for four iodine species, including I^−^, IO_3_^−^, I_3_^−^, and I_2_, was explored, and MOF‐monoAg reaches the highest removal rate for I^−^ ions of 98.8% (**Figure** **3**a). In addition to I^−^ ions, MOF‐monoAg also exhibits relatively high adsorption abilities for the other iodine species, indicating its potential for the recovery of other iodine species. The removal kinetics reveal that MOF‐monoAg rapidly adsorbs I^−^ ions during the first 30 min, achieving removal efficiencies of 99.5%, 99.1% and 98.8% eventually at initial I^−^ ion concentrations of 10, 50, and 100 ppm, respectively (Figure S14, Supporting Information). To evaluate the adsorption performance of MOF‐monoAg, batch adsorption experiments were carried out in aqueous I^−^ ion solutions. MOF‐monoAg reaches adsorption equilibrium rapidly within 15 min and finally achieves the equilibrium I^−^ ion adsorption capacity of 161.84 mg g^−1^ from 150 ppm I^−^ ion solution (Figure 3b). Fitting of the adsorption kinetics indicates that the pseudo‐second‐order model can describe the adsorption process better, suggesting that I^−^ ions are primarily adsorbed via chemical adsorption (Table S2, Supporting Information). Based on the silver content in MOF‐monoAg and assuming that each Ag^+^ site binds one I^−^ ion, the ratio of the apparent adsorption capacity to the theoretical maximum adsorption capacity of MOF‐monoAg for I^−^ ions is calculated to be 124%, suggesting that the enhanced uptake is attributed to multi‐iodide binding at Ag^+^ sites (Table S3, Supporting Information).^[^ ^35^ ^]^ To explore the theoretical maximum adsorption capacity, the adsorption isotherm of MOF‐monoAg was analyzed in I^−^ ion solutions with concentrations ranging from 5 to 150 ppm (Figure S15, Supporting Information). Based on the adsorption isotherm, the adsorption behavior of MOF‐monoAg to I^−^ ions fits better with the Langmuir model, indicating that the adsorption of I^−^ ions is mainly governed by monolayer sorption (Table S4, Supporting Information). The theoretical maximum adsorption capacity of MOF‐monoAg for I^−^ ions is calculated as 175.5 mg g^−1^, which is close to the experimental result. This further confirms that the chemical adsorption sites on MOF‐monoAg are occupied by I^−^ ions with high efficiency. The comparison of the distribution coefficient (*K*_d_) of MOF‐monoAg with that of previously reported functional adsorbents for I^−^ ion shows that the *K*_d_ value of MOF‐monoAg is 1.16 x 10^4^ mL g^−1^, noticeably higher than those of the other reported adsorbents (Figure 3c; Table S5, Supporting Information). Furthermore, the adsorption rate of MOF‐monoAg for I^−^ ions reaches 10.48 mg g^−1^ min^−1^, which is also significantly faster than previously reported I^−^ ion adsorbents, including a rate ≈28 times faster than that of Ag‐loaded MIL‐101.^[^ ^36^ ^]^ The high adsorption capacity and fast adsorption rate are mainly attributed to the sufficient internal spaces for the efficient transport of I^−^ ions within the framework and the access of I^−^ ions to the functional Ag^+^ sites. Additionally, MOF‐monoAg exhibits high anti‐interference ability (Figure 3d). With the presence of 100 times coexisting interfering anions, including NO_3_^−^, SO_4_^2−^, H_2_PO_4_^−^, CO_3_^2−^, F^−^, Cl^−^, and Br^−^, the removal efficiency for I^−^ ions is only slightly decreasing.

### 2.3 Removal Ability of MOF‐monoAg for I^−^ Ions in Simulated Nuclear Wastewater

To further validate the application potential of MOF‐monoAg for treating aqueous I^−^ ion pollution, simulated nuclear wastewater containing I^−^ ion was used. The simulated Hanford effluent, which contains 20 ppm of I^−^ ions along with various other anions, including NO_3_^−^, NO_2_^−^, Cl^−^, and SO_4_^2−^ at concentrations approximately hundreds of times higher than that of I^−^ ions, was prepared (Table S6, Supporting Information). The removal rates of MOF‐monoAg for I^−^, NO_2_^−^, SO_4_^2−^, Cl^−^, and NO_3_^−^ ions from the simulated Hanford wastewater are 98.7%, 3.7%, 3.0%, 0.32%, and 0.13%, respectively (Figure 3e). The removal rate of I^−^ ions is much higher than that of other anions, highlighting the exceptional selectivity and anti‐interference ability of MOF‐monoAg for I^−^ ions. In addition, evaluating the recyclability of the material is crucial to assessing its practical applicability. The successful elution of iodide was evidenced by EDS mapping, which shows the disappearance of iodine element and the appearance of chlorine element after desorption, while MOF‐monoAg retains its characteristic octahedral morphology (Figures S16, Supporting Information). After 5 cycles of adsorption‐desorption, MOF‐monoAg could still remove over 96.1% of I^−^ ions (Figures S17, Supporting Information). These results confirm the significant application potential of MOF‐monoAg for treating I^−^ ion pollution.

### 2.4 Recovery Ability of MOF‐monoAg for Iodine in Seawater

With its vast reserves, the effective recovery of iodine from seawater is highly possible to meet the long‐term demand for iodine resources. Therefore, the recovery abilities of MOF‐monoAg for iodine in both simulated seawater and natural seawater were determined. MOF‐monoAg exhibits rapid removal kinetics at a trace I^−^ ion concentration of 60 ppb, removing 82.1% of I^−^ ions within the first 2 min and achieving an equilibrium removal efficiency of 99.0%, indicating the high removal efficiency of MOF‐monoAg for I^−^ ions at seawater‐level iodine concentrations (Figure S18, Supporting Information). In simulated seawater, MOF‐monoAg realizes a maximum adsorption capacity of 148.0 mg g^−1^ with the initial I^−^ ion concentration of 150 ppm and the adsorbent dosage of 10 mg L^−1^ (Figure S19, Supporting Information). This result proves the promising potential of the adsorbent for iodine resource extraction from natural seawater, and thus, the natural seawater was collected to determine the practical iodine extraction capacity. When applied to natural seawater with the dosage of 0.1 mg L^−1^, MOF‐monoAg achieves a high extraction capacity of 114.2 mg g^−1^ for iodine, which is ≈50 times higher than the content of iodine found in seaweed *Laminaria japonica* widely used for industrial iodine extraction from seawater (Figure 3f,g).^[^ ^37^, ^38^ ^]^ Moreover, the extraction rate of iodine from seawater by MOF‐monoAg is more than 2000 times higher than that of seaweed, which has a growth cycle of six months. After iodine extraction, the concentrations of Ag^+^ remain below the detection limit, and BF_4_^−^ increases by only 0.40% in the tested seawater, thereby excluding secondary contamination during the extraction process (Table S7, Supporting Information). The exceptional iodine extraction performance of MOF‐monoAg in natural seawater empowers it as a highly potential adsorbent for utilizing seawater iodine resources.

### 2.5 Removal Ability of MOF‐monoAg for I^−^ Ions in Iodine‐Exposed Drinking Water

**Figure 4:**
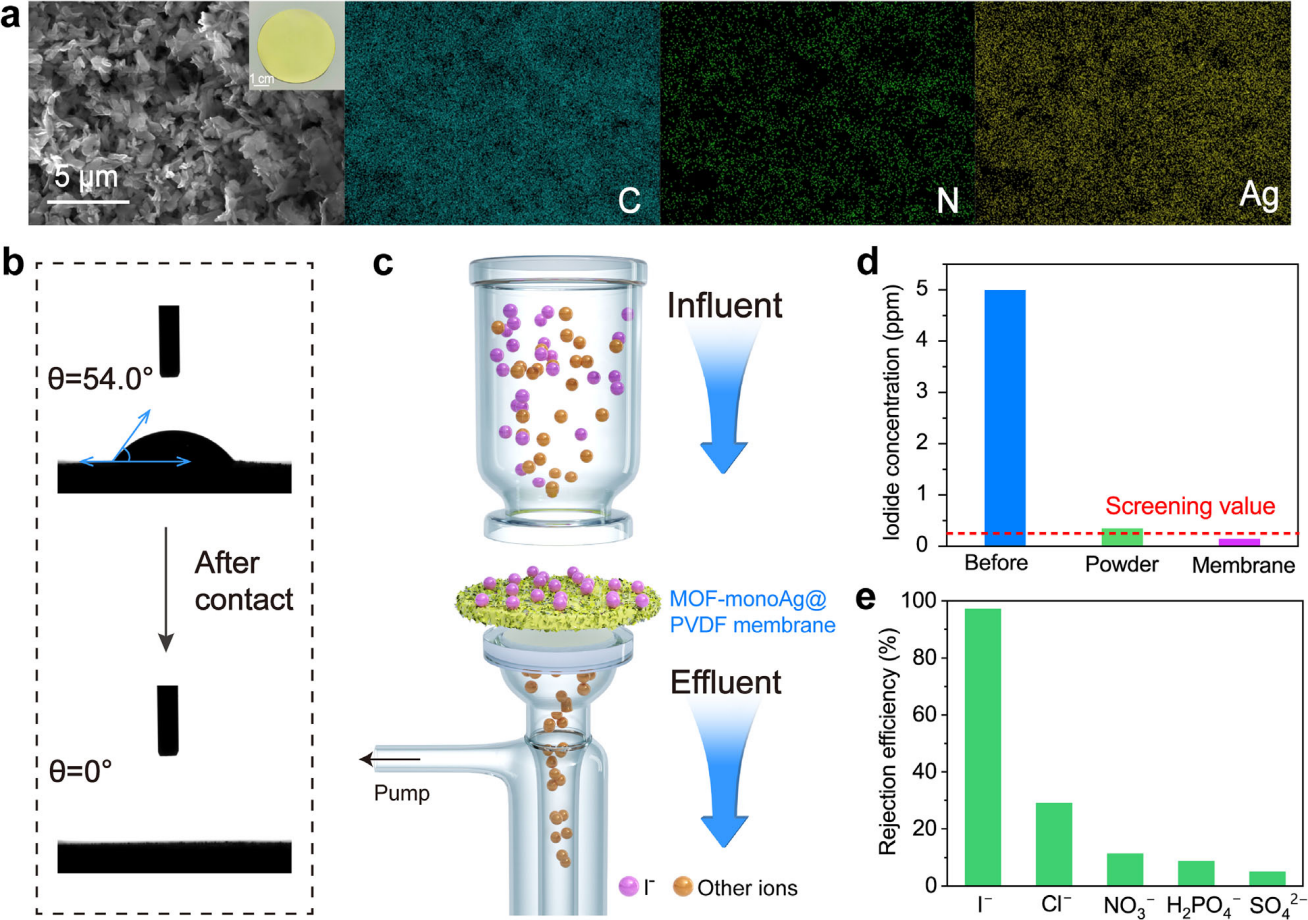
Rejection performance of DMFS based on the composite MOF‐monoAg@PVDF membrane. a) Morphology and corresponding EDS mapping of the composite membrane. Inset: digital photograph of the composite MOF‐monoAg@PVDF membrane. b) Water contact angles of the composite membrane during interaction with a water drop. c) Schematic representation of iodide‐polluted natural water purification by the composite membrane. d) Iodide concentration before and after being treated by MOF‐monoAg powder (dosage ratio: 1 mg mL^−1^) and DMFS. The screening threshold established by Canada for iodide in drinking water (0.24 ppm) is shown. e) The rejection efficiency for iodide ion and other anions through DMFS.

Iodine is commonly used for short‐term or emergency disinfection of drinking water due to its strong oxidation properties, which, however, may lead to excessive iodine exposure in the treated water.^[^ ^39^ ^]^ To address this issue, a composite MOF‐monoAg@PVDF membrane was fabricated for the effective purification of iodide‐exposed water (**Figure** **4**a). SEM imaging reveals that the pristine membrane exhibits uniformly distributed pores (Figure S20, Supporting Information). In contrast, after loading with MOF‐monoAg, the membrane surface exhibits stacked nanosheets with a uniform distribution of C, N, and Ag elements, as confirmed by the EDS analysis (Figure 4a). Water contact angle measurement shows that the composite MOF‐monoAg@PVDF membrane possesses a hydrophilic surface, as water can penetrate into the layer within merely 1.4 s, making it highly suitable for efficient water purification (Figure 4b). A DMFS was then developed using the composite membrane to remove iodide from iodine‐exposed water (Figure 4c; Figure S21 and Table S8, Supporting Information). The composite membrane demonstrates better performance compared to MOF‐monoAg powder, successfully reducing the iodide concentration to 0.14 ppm, which is below the established Canada drinking water threshold (Figure 4d). Notably, the composite membrane demonstrates a 97.2% rejection efficiency for I^−^ ion, while exhibiting much lower rejection efficiencies for Cl^−^ (29.1%), NO_3_^−^ (11.3%), H_2_PO_4_^−^ (8.7%), and SO_4_^2−^ (4.9%), indicating its high selectivity for iodide ions (Figure 4e). Furthermore, DMFS maintains a rejection efficiency of 85.6% even after ten treatment cycles, highlighting its stability under cyclic operation (Figure S22, Supporting Information). Thus, the DMFS based on the composite MOF‐monoAg@PVDF membrane holds significant potential for addressing iodide contamination in water caused by iodine‐based water disinfection.

### 2.6 Mechanism for Selective Adsorption of I^−^ Ions

**Figure 5:**
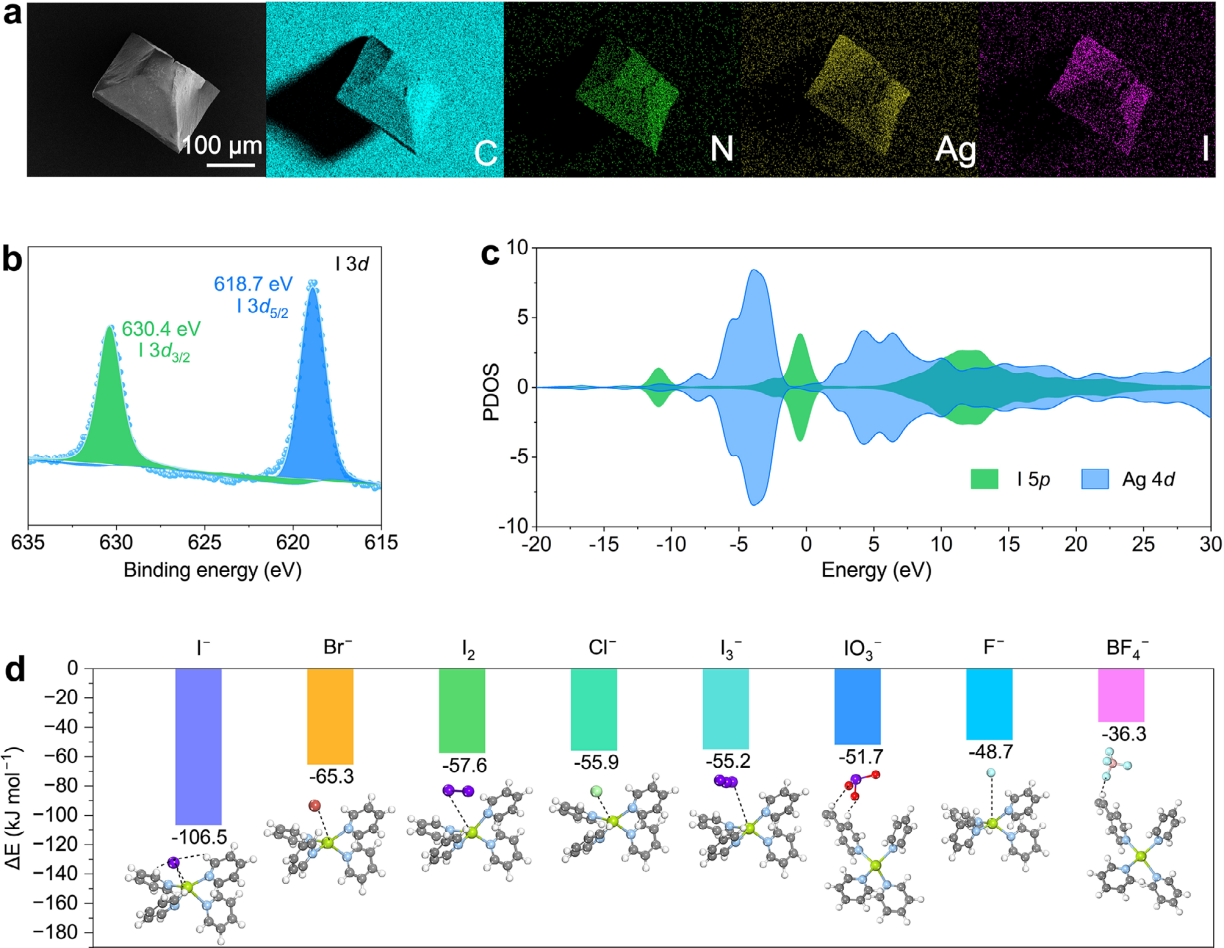
Adsorption mechanism of MOF‐monoAg to I^−^ ions. a) Morphology and corresponding EDS mapping of I^−^ ion‐loaded MOF‐monoAg. b) High‐resolution XPS spectrum of I 3*d* on MOF‐monoAg after I^−^ ions adsorption. c) PDOS for I 5*p* and Ag 4*d* orbitals in I^−^ ion‐loaded MOF‐monoAg. d) Binding energies of different iodine species, halide ions, and BF_4_^−^ ion on MOF‐monoAg.

The adsorption mechanism of MOF‐monoAg for I^−^ ions was explored in detail by analyzing I^−^ ion‐loaded MOF‐monoAg obtained after adsorption from I^−^ ion solution. The PXRD pattern of I^−^ ion‐loaded MOF‐monoAg reveals that MOF‐monoAg maintains its initial crystal structure after being used in I^−^ ion solution, with no characteristic reflections of AgI detected (JCPDS 09–0374), thus excluding the formation of this phase (Figure S23, Supporting Information). SEM‐EDS mapping analysis confirms the uniform distribution of iodine element on I^−^ ion‐loaded MOF‐monoAg, proving the successful adsorption of iodine (**Figure** **5**a). Transmission electron microscopy (TEM) and EDS mapping confirm the uniformity of the MOF‐monoAg surface without any deposition, while high‐resolution TEM image and fast Fourier transform patterns show lattice fringes indexed to the $\left(3\bar{2}\bar{4}\right)$ plane of MOF‐monoAg, all of which are consistent with the PXRD results (Figures S24 and S25, Supporting Information). After I^−^ ions adsorption, the BET surface area and pore volume decreased to 37.2 m^2^ g^−1^ and 0.09 cm^3^ g^−1^, respectively, indicating that the spaces within the framework are occupied by I^−^ ions (Figure S26, Supporting Information). FTIR analysis of I^−^ ion‐loaded MOF‐monoAg reveals a red shift in the C═N vibration peak, resulting from the binding of I^−^ ions by the Ag^+^ sites, which leads to the change in the electron environment of the nitrogen atoms in the C═N groups coordinating with the Ag^+^ sites (Figure S27, Supporting Information).^[^ ^40^ ^]^ Furthermore, the strength of the peak of BF_4_^−^ becomes weaker compared with that in the as‐synthesized MOF‐monoAg, proving the replacement of BF_4_^−^ ions by I^−^ ions. Electron paramagnetic resonance spectroscopy analysis reveals a prominent signal after the adsorption of I^−^ ions, indicating strong charge transfer between I^−^ ion and MOF‐monoAg (Figure S28, Supporting Information).^[^ ^41^ ^]^ The result of high‐resolution X‐ray photoelectron spectroscopy (XPS) spectrum of the I 3*d* region after I^−^ ions adsorption reveals the existence of the signal peaks at 630.4 eV (I 3*d*_3/2_) and 618.7 eV (I 3*d*_5/2_), further confirming the adsorption of iodine by MOF‐monoAg (Figure 5b). The high‐resolution XPS spectra of Ag 3*d* shows that the peaks of Ag 3*d*_3/2_ and Ag 3*d*_5/2_ shifts from 374.3 to 374.1 eV and 368.3 to 368.1 eV after the adsorption of I^−^ ions, which can be attributed to the share of electrons between I^−^ ion and Ag^+^ site, thus confirming the binding of I^−^ ions by Ag^+^ sites in the framework (Figure S29, Supporting Information).

To further elucidate the adsorption mechanism of I^−^ ions by MOF‐monoAg, the density functional theory (DFT) calculations were performed. The partial density of states (PDOS) of I^−^ ion and Ag^+^ site in I^−^ ion‐loaded MOF‐monoAg was further calculated to uncover their electron states (Figure 5c). The electron density distributions for the 4*d* orbital of Ag^+^ site and the 5*p* orbital of I^−^ ion are found to overlap around the Fermi level, suggesting the electron transfer between I^−^ ion and Ag^+^ site.^[^ ^42^, ^43^ ^]^ Compared to the isolated I^−^ ion, the calculated PDOS of I^−^ ion bound by MOF‐monoAg migrates toward lower energy levels, with a new broad peak emerging at the position of 12 eV, which proves that I^−^ ion tends to form a stable electronic state after being adsorbed by MOF‐monoAg (Figure S30, Supporting Information).^[^ ^44^ ^]^

The calculated charge density difference has also been calculated to provide a deeper understanding of the electron transfer behavior, and the result shows that a significant electron transfer can happen from I^−^ ion to Ag^+^ site, which is consistent with the results of the PDOS analysis (Figure S31, Supporting Information). I^−^ ion is calculated to be adsorbed onto the Ag^+^ site through the formation of Ag─I coordination bond with a length of 2.816 Å (Figure 5d; Figure S32, Supporting Information). Furthermore, three hydrogen bonds from the organic ligands are formed with the I^−^ ion, facilitating the binding of the I^−^ ion. Considering the high selectivity of MOF‐monoAg for I^−^ ion, the binding energies of MOF‐monoAg with different iodine species, halide ions, and BF_4_^−^ ion were also calculated. The highest binding energy of −106.5 kJ mol^−1^ is observed for the I^−^ ion and considerably exceeds that obtained for the BF_4_^−^ ion of −36.3 kJ mol^−1^ and other ions and different iodine species. The higher binding affinity of the skeleton for I^−^ ion is attributed to the synergistic effects of the specific interactions between Ag^+^ ion and I^−^ ion, as well as the assistance of the hydrogen bonding interaction, which is responsible for the superior removal efficiency and high selectivity for I^−^ ions of MOF‐monoAg for use in natural environments.

Based on the above experimental and computational results, the enhanced performance of MOF‐monoAg for I^−^ ions could be attributed to the rational distribution of Ag^+^ sites and the spatial structure of MOF‐monoAg. The high binding selectivity and affinity of the monodispersed Ag^+^ sites for I^−^ ions ensure the superior adsorption selectivity of MOF‐monoAg to I^−^ ions. The sufficient transport channels within the framework and the full exposure of the functional Ag^+^ sites contribute to its high adsorption capacity and fast adsorption rate for I^−^ ions. As a result, MOF‐monoAg exhibits significant potential for treating environmental iodine pollution and utilizing seawater iodine resources.

## 3 Conclusion

In summary, to overcome the challenges in recovering aqueous I^−^ ions, a novel cationic MOF adsorbent, MOF‐monoAg was synthesized by introducing monodispersed Ag^+^ sites. The high binding affinity and selectivity of the Ag^+^ sites for I^−^ ions contribute to the high adsorption specificity of the adsorbent. The monodispersed feature of the Ag^+^ sites combined with sufficient internal space benefits efficient transport and access of I^−^ ions to these functional sites, resulting in high adsorption capacity and fast adsorption rate. As a result, MOF‐monoAg realizes a high *K*_d_ value of 1.16 x 10^4^ mL g^−1^ and a fast adsorption rate of 10.48 mg g^−1^ min^−1^ for aqueous I^−^ ions. More importantly, it demonstrates an impressive I^−^ ions removal rate of 98.7% and a record iodine extraction capacity of 114.2 mg g^−1^ in simulated nuclear wastewater and natural seawater, respectively. The iodine extraction capacity and extraction rate of MOF‐monoAg in natural seawater significantly outperform those of seaweed, that widely used for industrial access to iodine resources from seawater. Furthermore, a DMFS is developed using the composite MOF‐monoAg@PVDF membrane and performs high rejection efficiency and selectivity for I^−^ ions in iodine‐exposed water, making it suitable for being utilized in the treatment of iodine‐exposed water contamination. The findings of this study inspire new directions for the recovery of aqueous I^−^ ions and will contribute to the sustainable access to seawater iodine resources and the efficient management of iodine pollution in nuclear wastewater and drinking water.

## Conflict of Interest

The authors declare no conflict of interest

## Supporting information



Supporting Information

## Data Availability

The data that support the findings of this study are available from the corresponding author upon reasonable request.
