# Supplementary material for: Metal–Organic Framework Featuring Monodispersed Silver Cation Sites for Highly Efficient and Selective Extraction of Aqueous Iodide Anions
Source: Adv Sci (Weinh). 2025 Oct 30;13(4):e17224. doi: 10.1002/advs.202517224 (PMC12822468; doi:10.1002/advs.202517224)
Supplement: Supplementary file 1 — Supporting Information [file ADVS-13-e17224-s001.pdf]

Supporting Information

Metal–Organic Framework Featuring Monodispersed Silver Cation Sites for Highly Efficient and Selective Extraction of Aqueous Iodide Anions

Xuewen Cao,[a] *Jiacheng Zhang,*[a] *Jinjiao Pan,*[a] *Yan Li,*[a] *Yue Ma,*[a] *Xinfeng Du,*[a] *Lijuan Feng,*[a] *Boyang Huang,*[b] *Yihui Yuan,**[a] *Liang Mao,**[b] *Ning Wang,**[a] *Abdullah M. Al-Enizi,*[d] *Ayman Nafady,*[d] *Shengqian Ma**[c]

[a] X. Cao, J. Zhang, J. Pan, Y. Li, Dr. Y. Ma, Dr. X. Du, Dr. L. Feng, Prof. Y. Yuan, Prof. N. Wang
State Key Laboratory of Marine Resource Utilization in South China Sea, Hainan University, Haikou 570228, P. R. China
E-mail: wangn02@foxmail.com (N. Wang), yuanyh@hainanu.edu.cn (Y. Yuan)

[b] Dr. B. Huang, Prof. L. Mao
State Key Laboratory of Pollution Control and Resource Reuse, School of the Environment, Chemistry and Biomedicine Innovation Center, Nanjing University, Nanjing 210093, P. R. China
E-mail: lmao@nju.edu.cn (L. Mao)

[c] Prof. S. Ma
Department of Chemistry, University of North Texas, Denton, TX, 76201 USA
E-mail: Shengqian.Ma@unt.edu (S. Ma)

[d] Prof. A. M. Al-Enizi, Prof. A. Nafady
Department of Chemistry, College of Science, King Saud University, Riyadh 11451, Saudi Arabia

Table of Contents

Experimental Procedures ... 3
Materials and reagents ... 3
Characterizations ... 3
Synthesis of MOF-monoAg ... 4
Synthesis of the composite membrane ... 4
Adsorption experiments ... 4
Computation methods ... 6
Supporting Figures and Tables ... 8
References ... 48

Experimental Procedures

Materials and reagents

1,1,2,2-Tetrakis(4-((E)-2-(pyridin-4-yl)vinyl)phenyl)ethene (TPVPE) was purchased by Jilin Chinese Academy of Sciences-Yanshen Technology Co., Ltd. NaOH, HCl, and $AgBF_4$ were purchased from Shanghai Macklin Biochemical Technology Co., Ltd. Sodium salts including NaI, NaBr, NaCl, NaF, $NaNO_3$, $NaNO_2$, Na_2HPO_4, Na_2CO_3, $NaHCO_3$ and Na_2SO_4 and the iodide standard solution (1mg ml^{-1} in water) were purchased from Aladdin Bio-chemical Technology Co, Ltd. Ammonium Hydroxide ($NH_3{\cdot}H_2O$, Spectrographic grade), ethanol (EtOH), acetonitrile (CH_3CN), dimethyl sulfoxide (DMSO), dichloromethane (CH_2Cl_2), and methanol (MeOH) were procured from Shanghai Titan Scientific Co., Ltd. The commercial PVDF membranes (50 mm, 0.22 μm) were purchased from Haining Delv Technology Co., Ltd. All chemicals and solvents used in this study were analytically pure and used without further purification.

Characterizations

Single-crystal X-ray diffraction data was collected using a diffractometer with a Turbo X-ray Source of Mo Kα radiation (λ = 0.71073 Å) at 193.0 K (SC-XRD, D8 Venture, Bruker, Germany). Powder X-ray diffraction patterns of as-synthesized MOF-monoAg were obtained on a Rigaku Smart Lab diffractometer by using a Cu Kα radiation source at a scanning rate of 2° min^{-1} for a 2θ range of 5° to 40° (PXRD, Smartlab-9KW, Rigaku, Japan). The morphology and element distribution of the material were examined by a field-emission scanning electron microscope (SEM, S-4800, HITACHI, Japan) equipped with an energy-dispersive X-ray spectroscopy (EDS) instrument. The microstructure, elemental mapping, and atomic-scale lattice fringes of the material were characterized using a transmission electron microscope (TEM, Talos F200X, FEI, USA) equipped with an EDS detector. The functional groups of the material were examined by a Fourier-transform infrared spectrometer (FTIR, LR 64912C, Perkin-Elmer, USA) by the KBr pellet method. N_2 adsorption–desorption isotherms were collected at 77 K using an automatic analyzer after the samples had been degassed at 80 °C under vacuum for 12 h (ASAP 2460, Mike, USA). The measurement of the element electron binding energy was carried out on the X-ray photoelectron spectrometer (XPS, Thermo ESCALAB 250Xi Thermo, USA) with an Al Kα source. Electron paramagnetic resonance spectra were carried out on a spectrometer (EPR, A300-10/12, Bruker, Germany). The content of C and N of the material was analyzed using an elemental analyzer (EA, UNICUBE, Elementar, Germany). The concentrations of Ag^+ and I^- were studied on an inductively coupled plasma mass spectrometer (ICP-MS, Perkin Elmer NexION 300, Perkin Elmer, USA), and the ion concentrations

were calculated from a suitable calibration curve ($R^2 \geq 0.9999$**). The concentrations of co-existing anions, including NO_3^-, NO_2^-, Cl^-, SO_4^{2-}, $H_2PO_4^-$, and BF_4^- , were analyzed by an ion chromatograph (IC, DIONEX AQUION RFIC, Thermo Fisher Scientific, USA).

Synthesis of MOF-monoAg

To synthesize MOF-monoAg, 9.3 mg organic ligand TPVPE and 10.2 mg metal nodes donor $AgBF_4$ were dissolved in the mixture solvent CH_3CN/DMSO (6 mL, v/v = 5/1) in a 10 mL glass bottle. The solution was heated at 90 °C for 48 h. The collected yellow crystals were washed with H_2O and MeOH several times and dried in air (Yield: 78%, based on $AgBF_4$). The phase purity of MOF-monoAg was checked by PXRD analysis. [CCDC number of 2389331 contains the supplementary crystallographic data for this paper. These data can be obtained free of charge from The Cambridge Crystallographic Data Centre via www.ccdc.cam.ac.uk/data_request/cif.]

Synthesis of the composite membrane

The pristine PVDF membrane was soaked in EtOH for 2 h in order to eliminate the attachments on the surface, followed by washing with deionized water and drying at room temperature for later use. In a 100 mL capped vial, 24.9 mg $AgBF_4$ and 1mL pyridine were added to 15 mL MeOH. 18.5 mg TPVPE dissolved in a mixture of CH_3CN, CH_2Cl_2, and MeOH totaling 20 mL was slowly dripped into the silver salt solution under ultrasound (v/v/v =9/6/5). Then, the pretreated PVDF membrane was immersed in this mixed solution at 50 °C using a constant temperature shaker for 48 h. The final yellow membrane was washed with MeOH and H_2O and dried in air.

Adsorption experiments

To evaluate the water stability of MOF-monoAg, 10 mg of MOF-monoAg was immersed in 10 mL of deionized water with varied solution conditions for 12 h, after which the solid was separated and the Ag^+ concentration in the supernatant was determined by ICP-MS. The leaching rate (R_L%) of Ag^+ from MOF-monoAg was calculated by the following equation (1):

$$R_{\mathrm{L}}\% = \frac{c_{\mathrm{s}}}{c_{\mathrm{d}}} \qquad (1)$$

where R_L% is the leaching rate, c_s (mg L^{-1}) is the concentration of Ag^+ in the solution after immersing materials, and c_d (mg L^{-1}) is the corresponding concentration when MOF-monoAg is completely decomposed, which is determined to be 109.82 ppm by ICP–MS.

All batch adsorption experiments were conducted at 25 °C with pH value of 7 with magnetic stirring unless otherwise noted. For four iodine species adsorption experiments, 5 mg MOF-monoAg was soaked in 5 mL of 100 ppm I^-, IO_3^-, I_3^-, and I_2 solutions for 6 h, respectively. For the I^- ions adsorption kinetics experiment, 5 mg MOF-monoAg was added to 500 mL solution containing 150 ppm I^- ion. The I^- ion solution was collected at each pre-set treated time. For the I^- ions adsorption isothermal experiment, the initial concentration range of I^- ion was set from 5 ppm to 150 ppm, and 5 mg MOF-monoAg was added to 500 mL of the above-described I^- ion solutions. For anion competition adsorption experiments, 5 mg MOF-monoAg was exposed to 5 mL of 10 ppm I^- ion solution mixing with 10- to 100-fold equimolar of competition anions (NO_3^-, SO_4^{2-}, $H_2PO_4^-$, CO_3^{2-}, F^-, Cl^-, and Br^-). The residual concentration of I^- ion was determined by ICP-MS after filtering with a 0.22 μm aqueous nylon filter membrane. The adsorption capacity (q_e) and the removal rate ($R_{\%}$) for I^- ions were calculated by the subtraction method, as instructed by equations (2-3):

$$q_e = \frac{(c_0 - c_e)V}{m} \quad (2)$$

$$R_{\%} = \frac{c_0 - c_e}{c_0} \times 100\% \quad (3)$$

where c_0 (mg L^{-1}) is the initial concentration of I^- ion and c_e (mg L^{-1}) is the concentration of I^- ion at adsorption equilibrium. V (mL) is the volume of the solution and m (mg) is the amount of the adsorbent.

To determine the rate-dominating step in the adsorption process, the data of I^- ions adsorption kinetics on MOF-monoAg was further fitted by the pseudo-first-order model and pseudo-second-order model with the following equations (4-5):

$$\lg(q_e - q_t) = lg(q_e) - \frac{k_1 t}{2.303} \quad (4)$$

$$\frac{t}{q_t} = \frac{1}{k_2 q_e^2} + \frac{t}{q_e} \quad (5)$$

where q_t (mg g^{-1}) denotes the adsorption capacity at time t, while q_e (mg g^{-1}) denotes the equilibrium adsorption capacity. k_1 and k_2 are defined as the constant of pseudo-first-order model and pseudo-second-order model.

The ratio of the apparent adsorption capacity to the theoretical maximum adsorption capacity of I^- ions is calculated as following equation (6):

$$A_{\%} = \frac{q_e}{1176 \times W_{Ag}\%} \quad (6)$$

where q_e (mg g^{-1}) denotes the equilibrium adsorption capacity, 1176 mg is the theoretical I^- ions adsorption capacity of 1 g of Ag, $W_{Ag}\%$ is the content of Ag in the framework.

To explore the interaction between adsorbent and I^- ion during the adsorption process, the data of I^- ions adsorption isotherm was fitted by the Langmuir model and the Freundlich model as described in equations (7-8):

$$q_e = \frac{q_{mL} K_L c_e}{1 + K_L c_e} \quad (7)$$

$$q_e = K_F c_e \hat{} (1/n_F) \quad (8)$$

where K_L (L mg^{-1}) and K_F (mg g^{-1}) (L mg^{-1})$^{1/n}$ are defined as the constants of the Langmuir and the Freundlich model. q_{mL} (mg g^{-1}) denotes the Langmuir maximum adsorption capacity. n_F indicates the adsorption affinity. q_e and c_e have the same meaning as Equation (2).

For I^- ions removal experiment in simulated wastewater, the composition of simulated Hanford wastewater was prepared according to a reported protocol and the concentration of each anion was provided in Table S6. A measured quantity of the simulated Hanford wastewater was pipetted into a glass bottle loaded with MOF-monoAg at a solid-liquid ratio of 2 g L^{-1} for 6 h at room temperature under magnetic stirring. After filtration through a 0.22 μm aqueous nylon filter membrane, the residual concentrations of I^- ion and other anions were analyzed by ICP-MS and IC.

For the reusability test, 10 mg MOF-monoAg was treated with 10 mL I^- ion solution of 10 ppm at room temperature for 4 h. After filtration, the iodide-loaded solids were regenerated by desorption in 5 M NaCl solution. Subsequently, the regenerated materials were utilized in further adsorption tests.

For I^- ion removal efficiency under seawater-level iodine concentrations, 10 mg MOF-monoAg was added to 10 mL I^- ion solution of 60 ppb at room temperature. The I^- ion solution was collected at each pre-set treated time. For I^- ion adsorption in simulated seawater, simulated seawater was prepared by dissolving sodium chloride (438.607 mM) into I^- ion solution. 5 mg MOF-monoAg was added to 500 mL simulated seawater containing different I^- ion concentrations varying from 1 ppm to 150 ppm under room temperature with 6 h magnetic stirring. For the iodine recovery experiment using natural seawater, natural seawater was collected from the west coast of Haikou City, Hainan Province, China. The iodine concentration in the natural seawater was measured to be ~58 ppb while the concentration of I^- ion and IO_3^- ion was 23 ppb and 35 ppb, respectively. 10 mg MOF-monoAg was immersed in 100 L of natural seawater with a flow rate of 5 L h^{-1} for seawater fluid under room temperature. The residual concentration of iodine and iodine recovery ability of MOF-monoAg were determined by ICP-MS and calculated by equation (2).

Computation methods

All density functional theory (DFT) calculations were carried out using the CP2K code.[1] All calculations employed mixed Gaussian and plane-wave basis sets. Core electrons were represented with norm-

conserving Goedecker-Teter-Hutter pseudopotentials and the valence electron wavefunction was expanded in a double-zeta basis set with polarization functions along with an auxiliary plane wave basis set with an energy cutoff of 450 eV.[2-5] The electrostatic potential (ESP) was calculated based on the ground state electron density. The generalized gradient approximation exchange-correlation functional of Perdew-Burke-Ernzerhof (PBE) was used to describe the electron interaction.[6] Each configuration was optimized by the Broyden-Fletcher-Goldfarb-Shanno (BFGS) algorithm with SCF convergence criteria of 1.0×10^{-6} au. To compensate the long-range van der Waals dispersion interaction, the DFT-D3 scheme with an empirical damped potential term was added to the energies obtained from exchange-correlation functional in all calculations.[7] The adsorption energy between MOF-monoAg and the different iodine species, halide ions, and BF_4^- ion can be calculated using the following equation (8):

$$\Delta E = E_{\mathrm{total}} - E_{\mathrm{MOF}} - E_{\mathrm{adsorbate}} \quad (8)$$

where E_{total} is the total energy of the framework structure with adsorbate. E_{MOF} and $E_{\mathrm{adsorbate}}$ are the energy of the framework and isolated adsorbate, respectively. According to this equation, a negative binding energy corresponds to a stable adsorption structure.

Supporting Figures and Tables

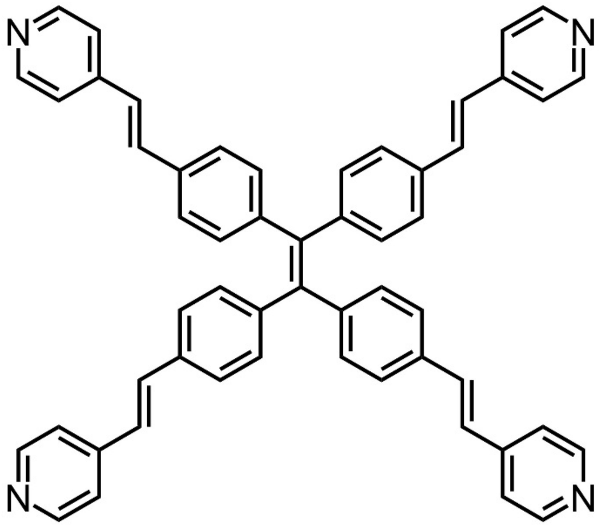

Figure S1. Chemical structure of the organic ligand 1,1,2,2-Tetrakis(4-((E)-2-(pyridin-4-yl)vinyl)phenyl)ethene (TPVPE).

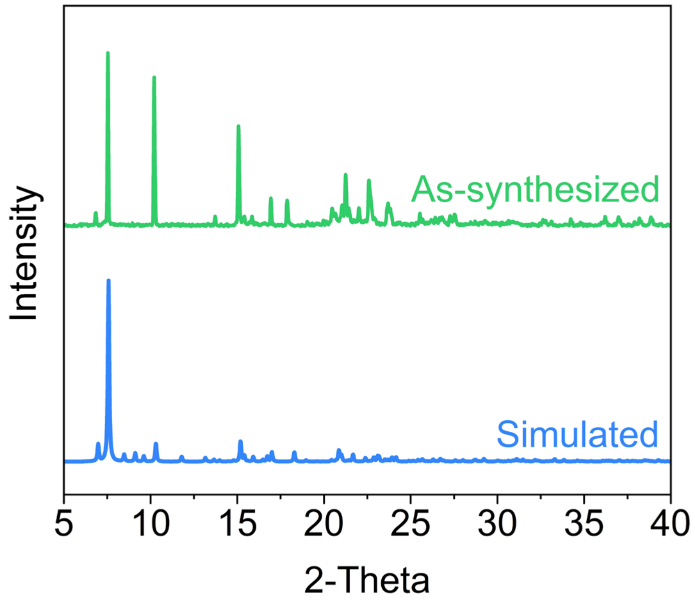

Figure S2. The as-synthesized and simulated PXRD patterns of MOF-monoAg.

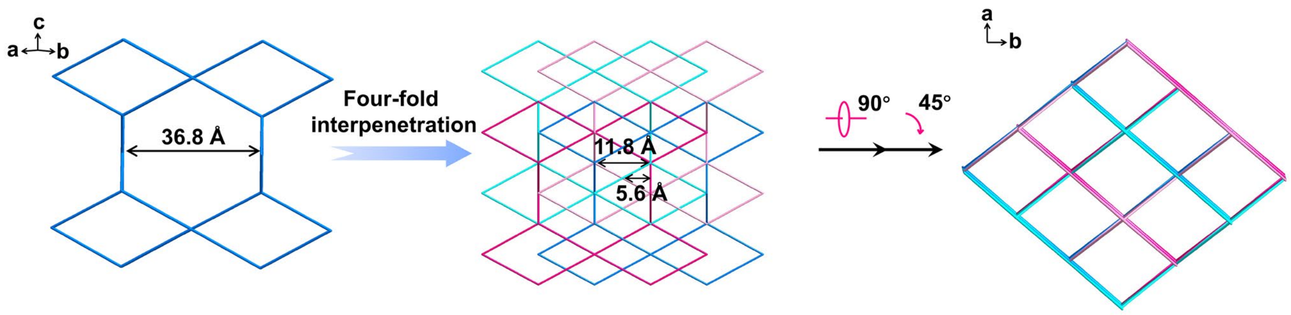

Figure S3. The simplified topological structure of four-fold interpenetration and the 1D channel windows of MOF-monoAg.

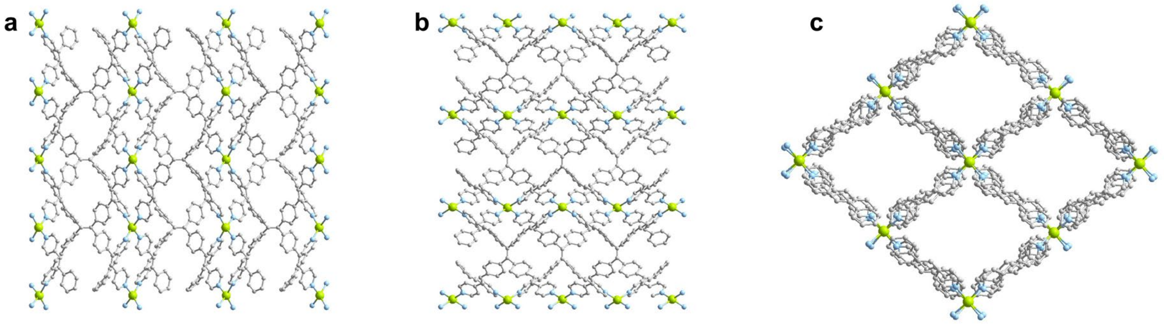

Figure S4. Packing structure of MOF-monoAg along (a) *a* axis, (b) *b* axis, and (c) *c* axis.

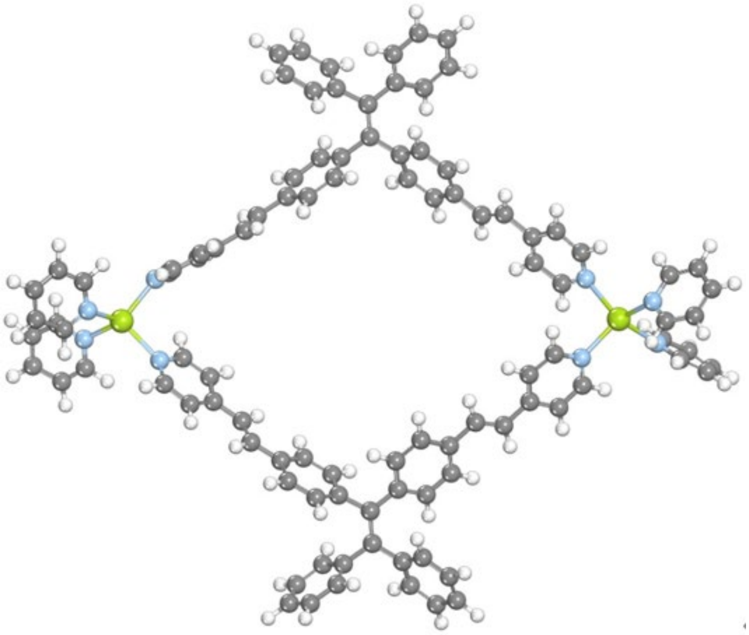

Figure S5. The optimized configuration of the partial structure in MOF-monoAg used for the DFT calculation.

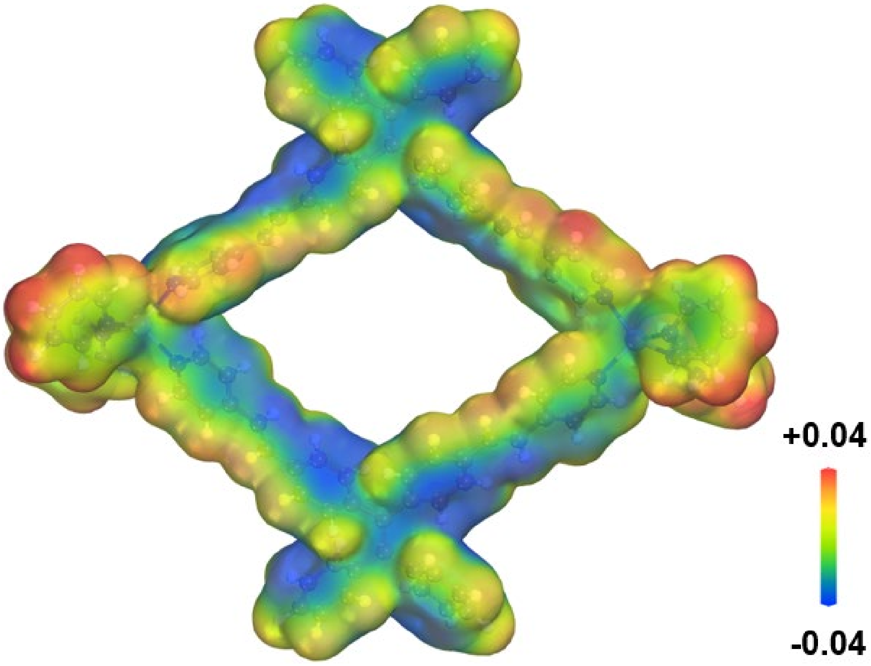

Figure S6. The corresponding electrostatic potential distribution (ESP) mapping of the optimized configuration of MOF-monoAg, where the red regions represent positive ionization energy and the blue regions represent negative ionization energy.

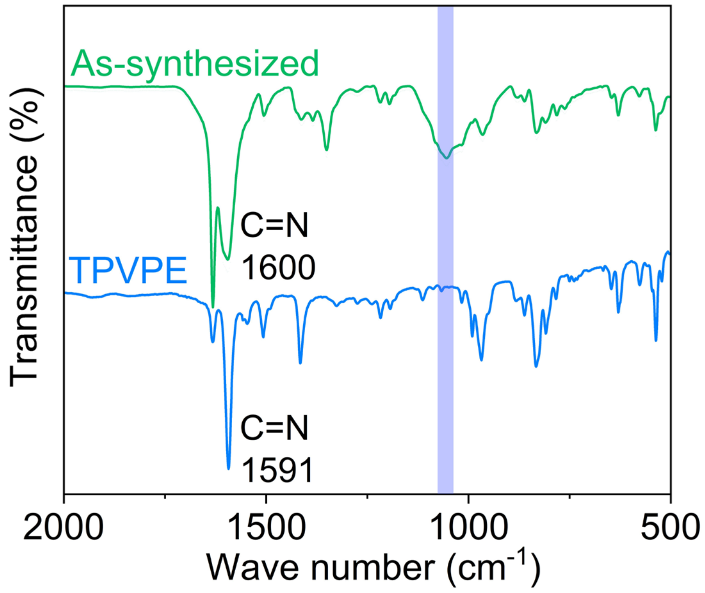

Figure S7. FTIR spectra of TPVPE and as-synthesized MOF-monoAg.

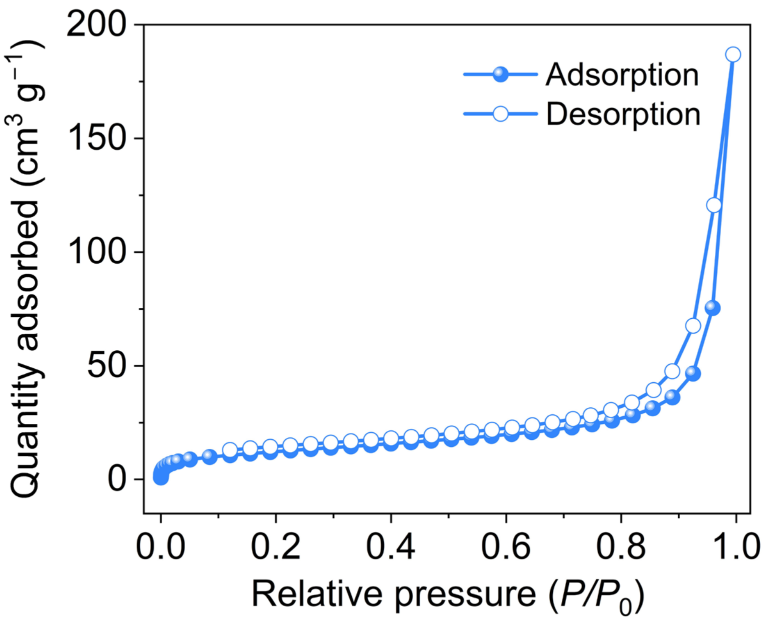

Figure S8. N_2 adsorption–desorption isotherm of MOF-monoAg.

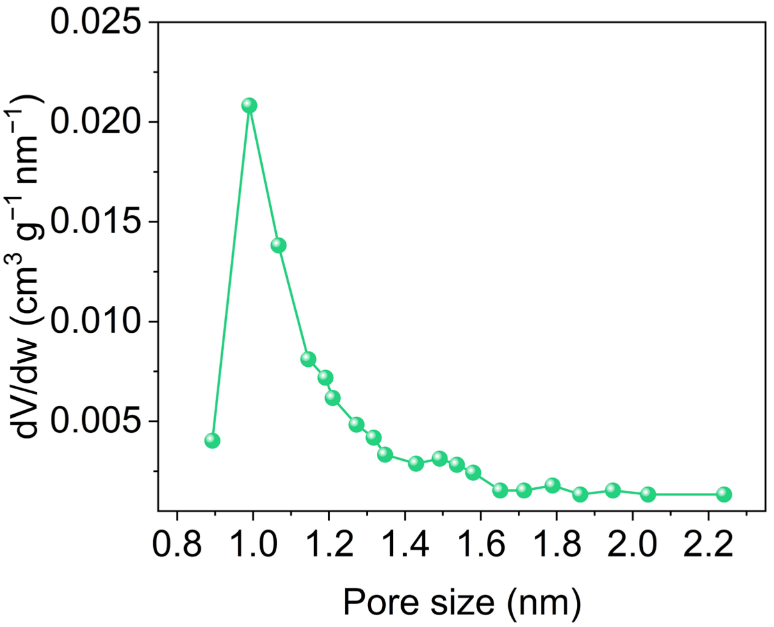

Figure S9. Pore size distribution of MOF-monoAg.

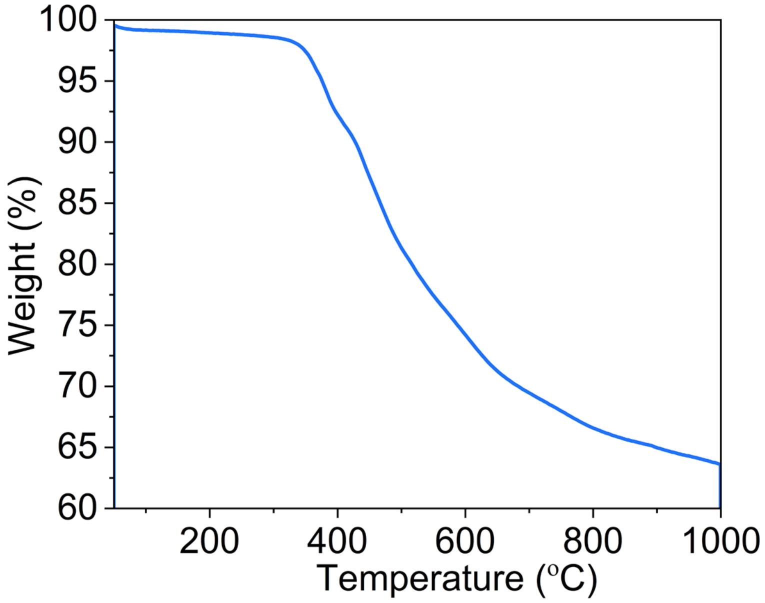

Figure S10. Thermogravimetric analysis of MOF-monoAg.

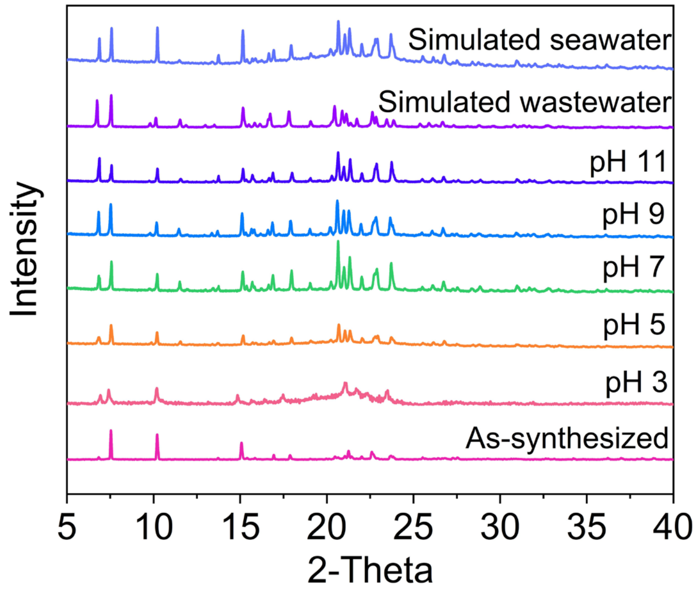

Figure S11. PXRD patterns of MOF-monoAg after immersion under different aqueous conditions.

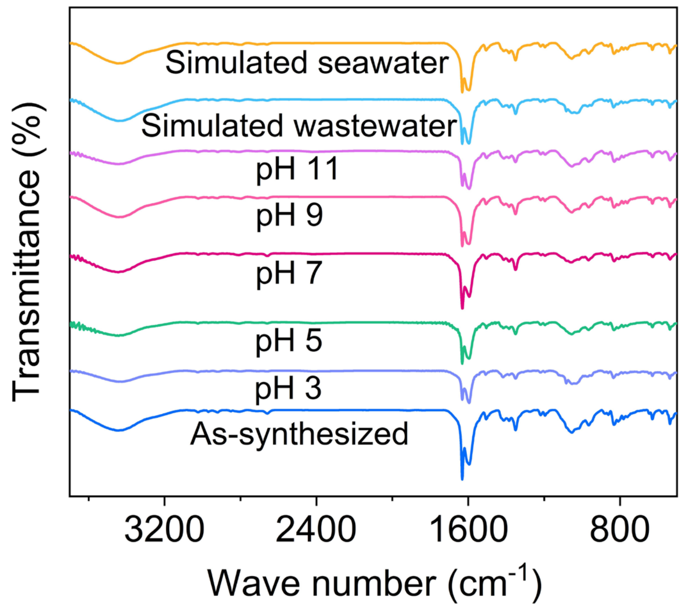

Figure S12. FTIR spectra of MOF-monoAg after immersion under different aqueous conditions.

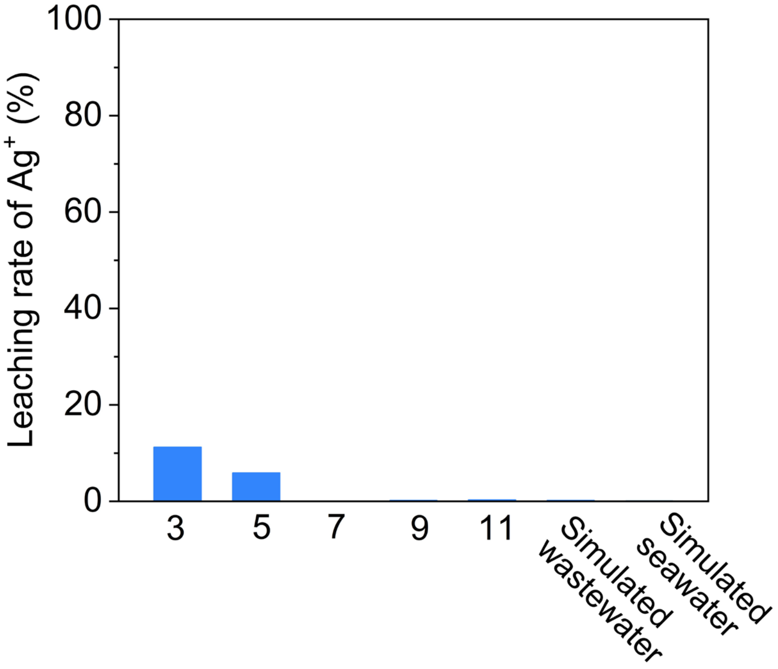

Figure S13. Leaching rate of Ag^+ from MOF-monoAg after immersion under different aqueous conditions.

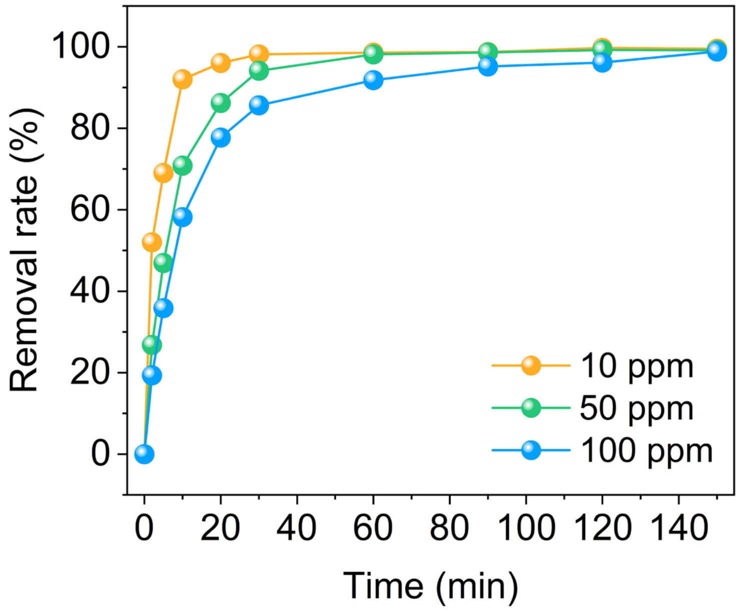

Figure S14. Removal kinetics of MOF-monoAg for I^- ions at initial iodide concentrations of 10 ppm, 50 ppm, and 100 ppm.

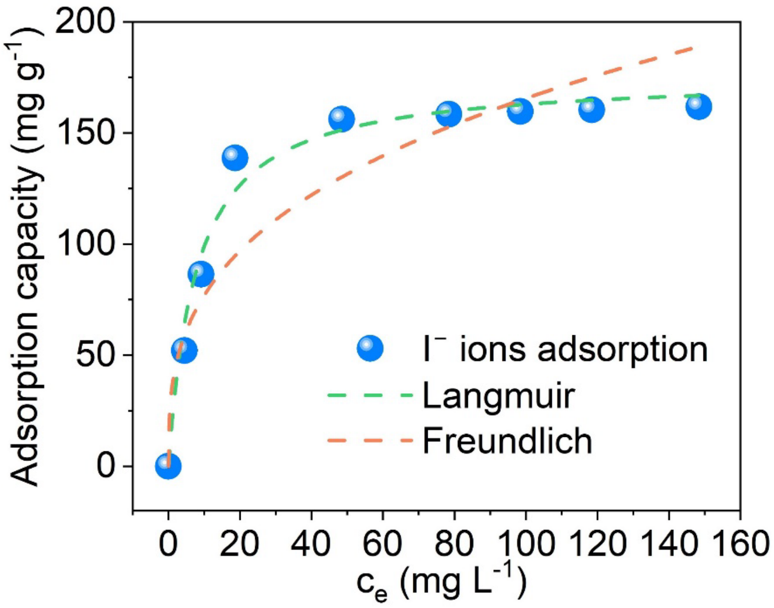

Figure S15. The adsorption isotherm of MOF-monoAg to aqueous I⁻ ions.

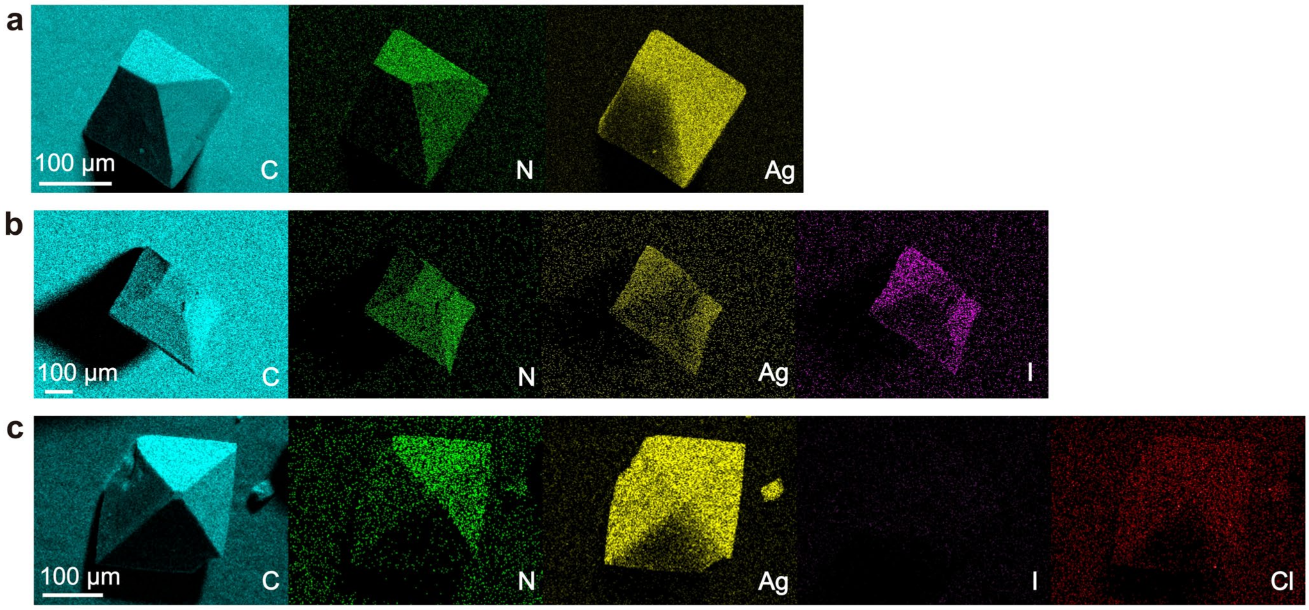

Figure S16. EDS mapping of (a) MOF-monoAg, (b) I⁻ ion-loaded MOF-monoAg, and (c) regenerated MOF-monoAg after 1st cycle of I⁻ ions adsorption.

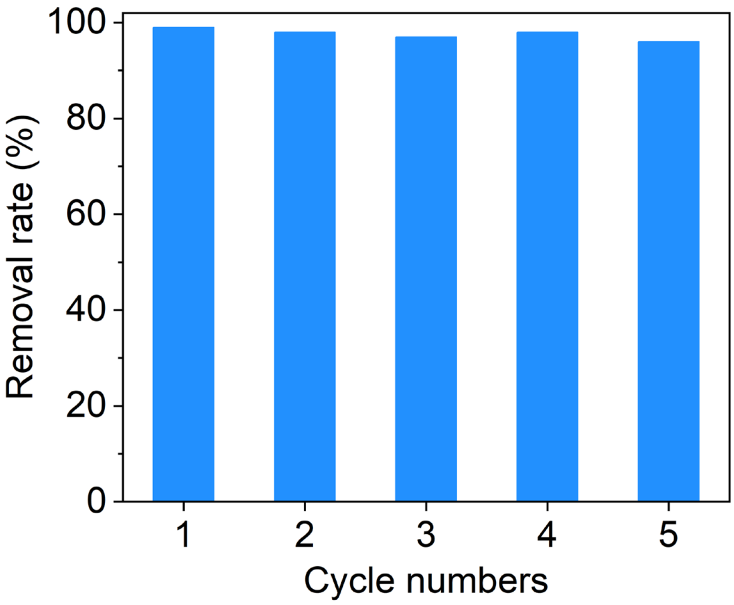

Figure S17. The reusability of MOF-monoAg for I^- ions removal.

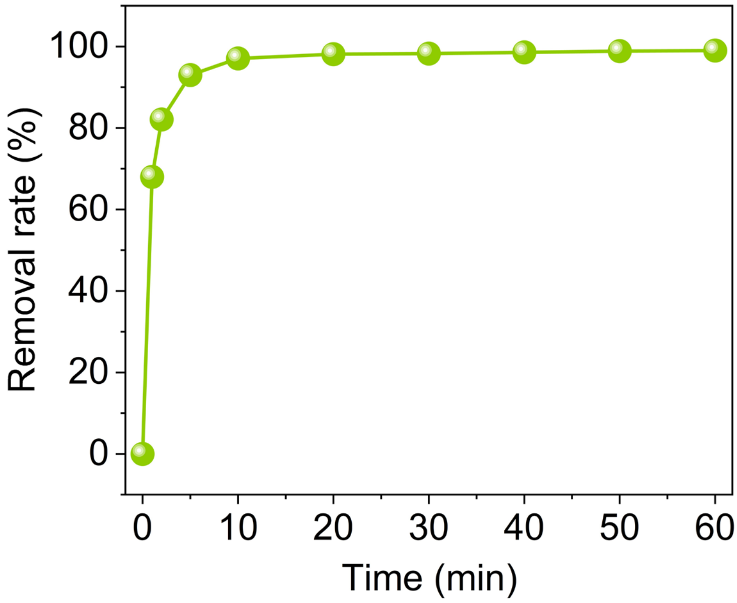

Figure S18. Removal kinetics of MOF-monoAg for I^- ions at an iodide concentration of 60 ppb.

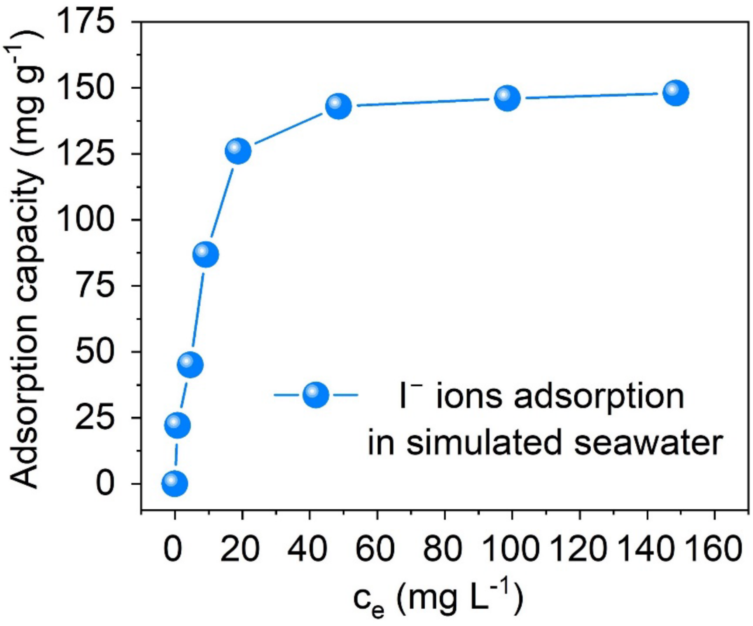

Figure S19. The adsorption isotherm of MOF-monoAg to I⁻ ions in simulated seawater.

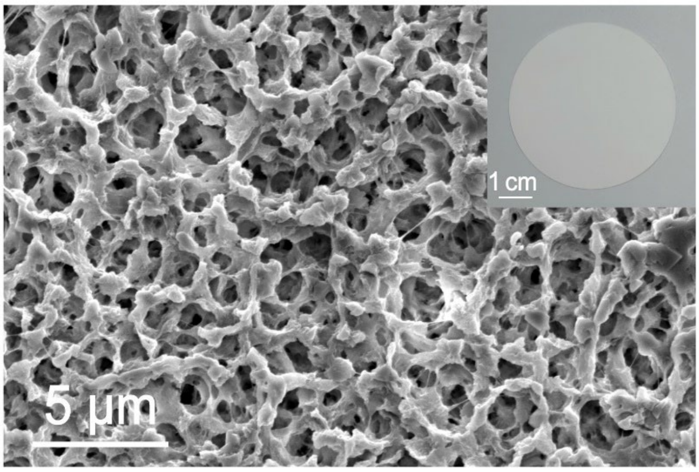

Figure S20. Morphology of pristine PVDF membrane. Inset: digital photograph of the pristine PVDF membrane.

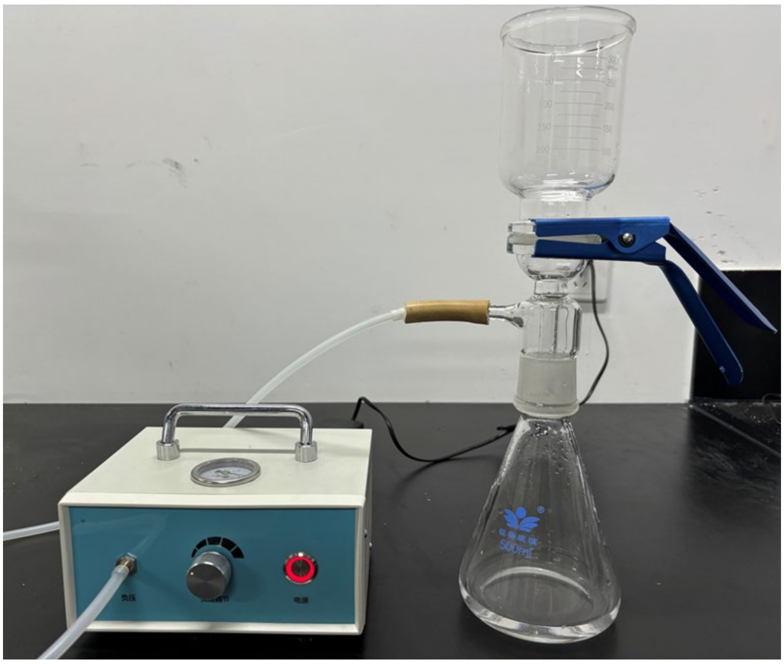

Figure S21. Diagram of the dynamic membrane filtration system for the purification of iodide-contaminated water. The iodide-exposed solution (10 mL each cycle) was filtered under a negative pressure of 0.1 bar.

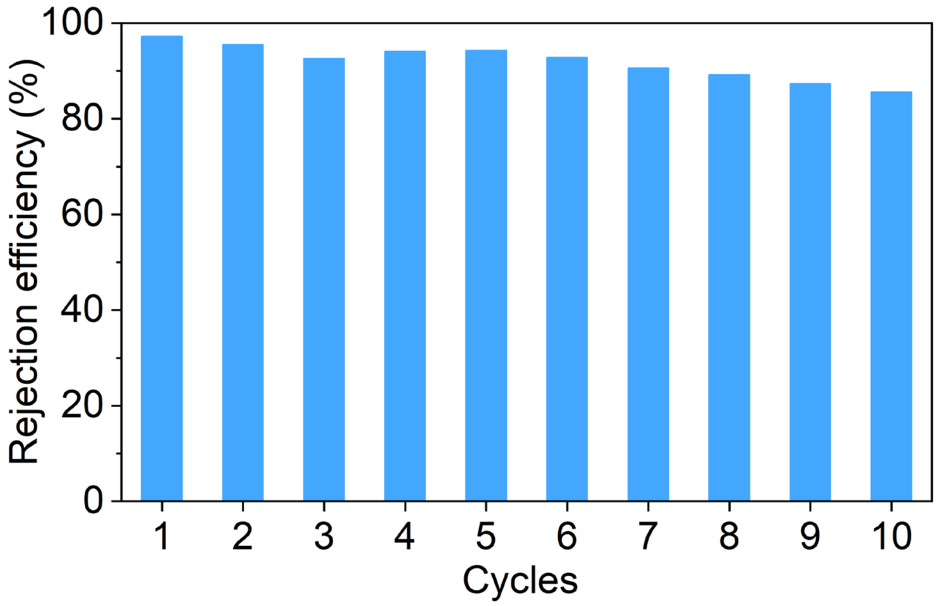

Figure S22. Recycling performance of the dynamic membrane filtration system for I^- ions rejection.

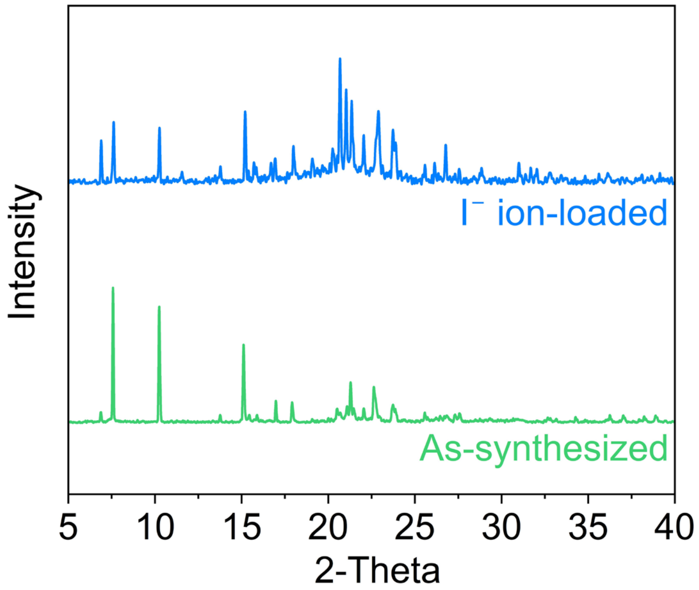

Figure S23. PXRD patterns of as-synthesized MOF-monoAg and I^- ion-loaded MOF-monoAg after being used in I^- ion solution.

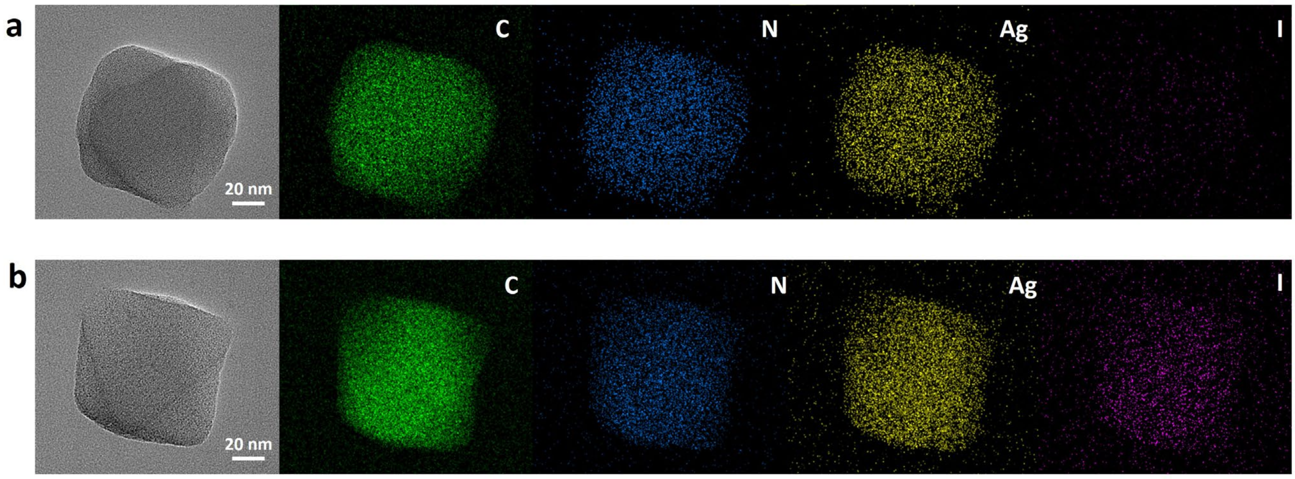

Figure S24. TEM images and corresponding EDS mapping on transmission electron microscopy mode of MOF-monoAg (a) before and (b) after I^- ions adsorption.

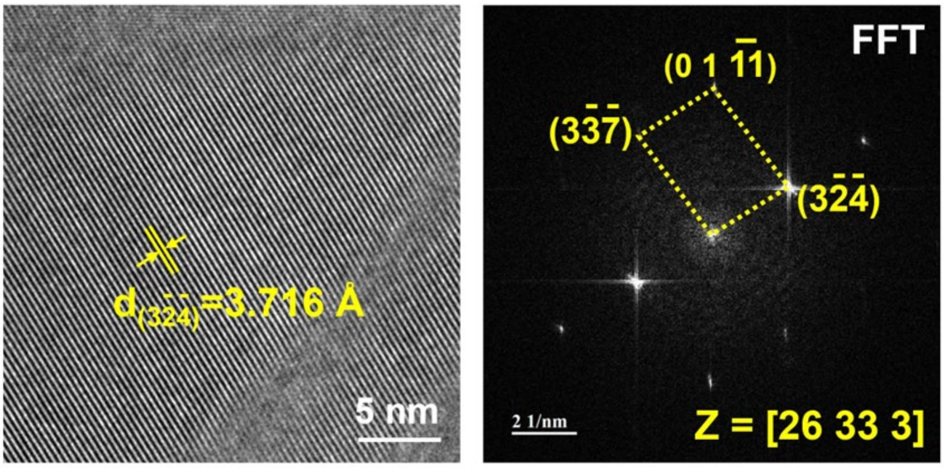

Figure S25. High-resolution TEM and fast fourier transform images of MOF-monoAg after I^- ions adsorption.

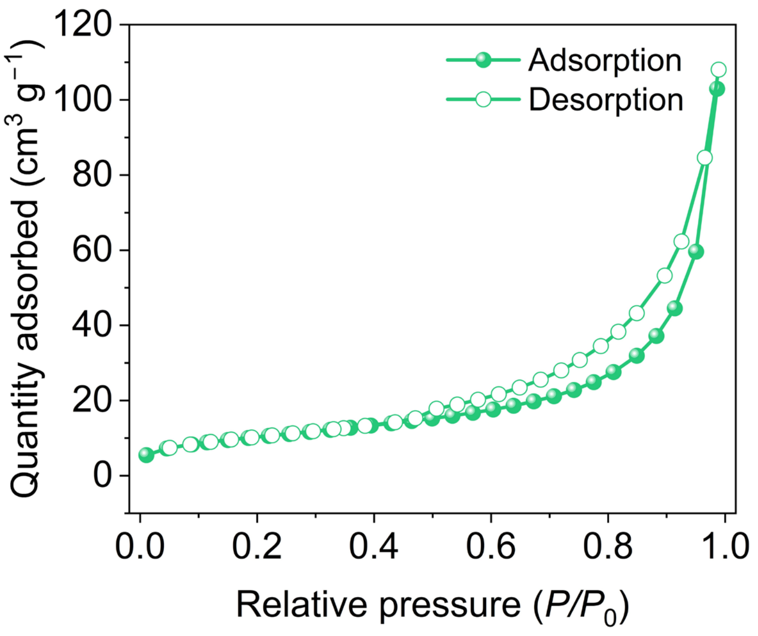

Figure S26. N_2 adsorption–desorption isotherm of MOF-monoAg after I^- ions adsorption.

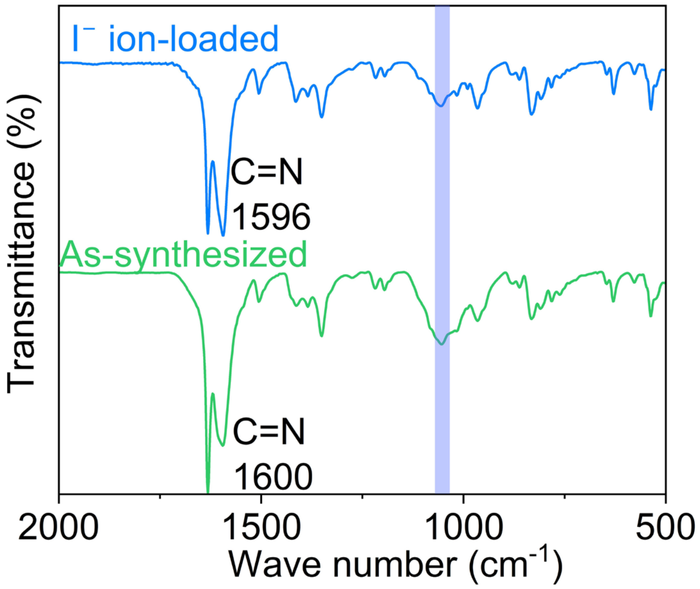

Figure S27. FTIR spectra of as-synthesized and I⁻ ion-loaded MOF-monoAg.

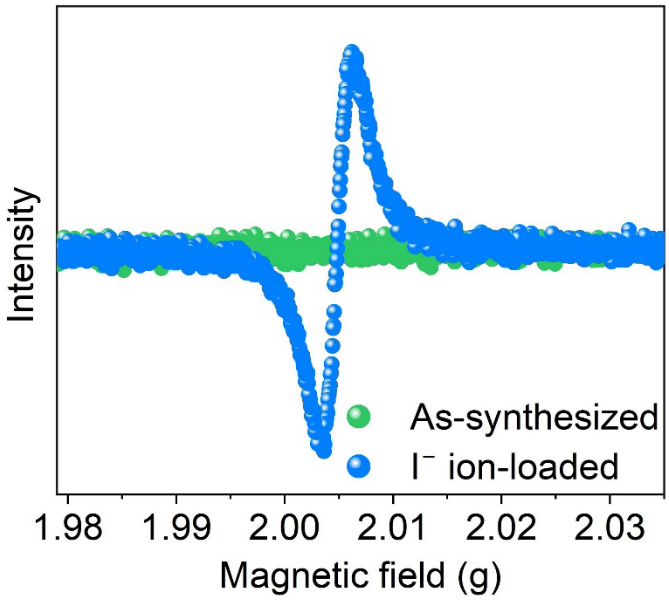

Figure S28. Electron paramagnetic resonance spectra of as-synthesized and I^- ion-loaded MOF-monoAg.

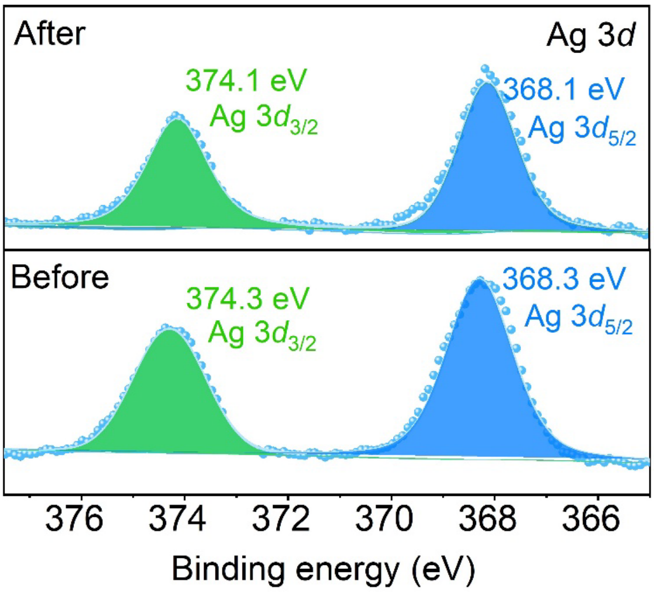

Figure S29. High-resolution XPS spectra of Ag 3*d* on MOF-monoAg before and after I^- ions adsorption.

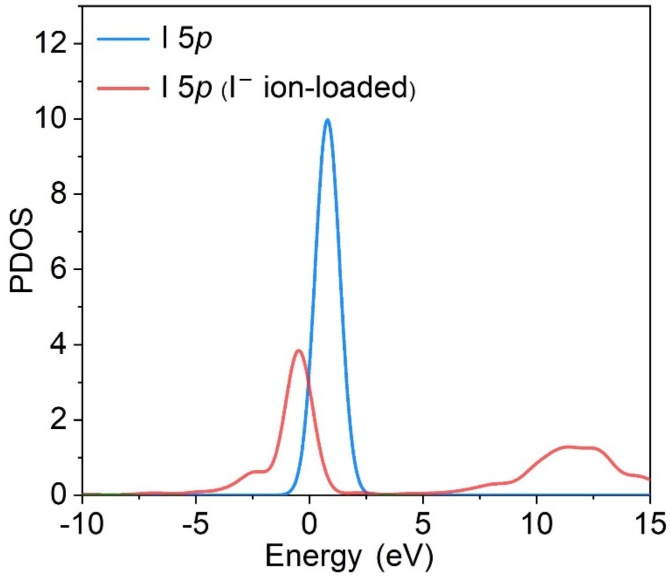

Figure S30. PDOS for I^- ion before and after binding on MOF-monoAg.

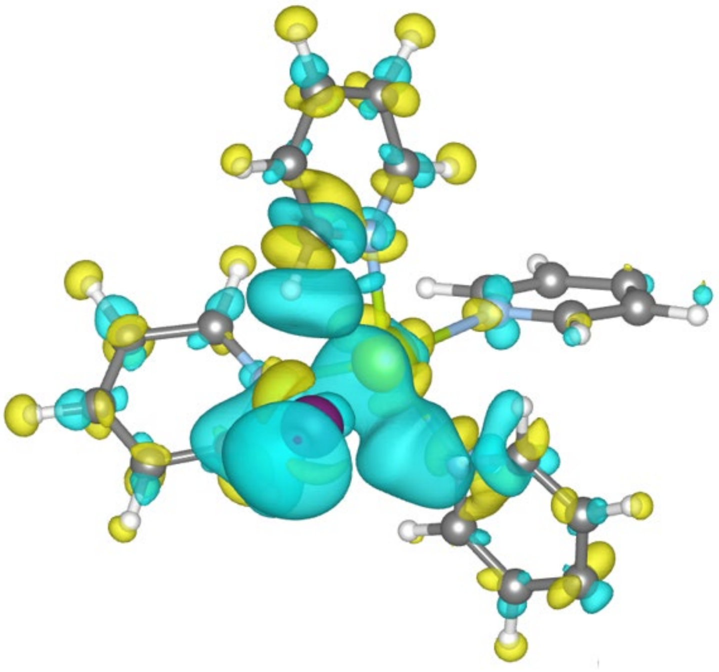

Figure S31. Charge density difference contour after I^- ion binding (electron accumulation and depletion regions are indicated by yellow and blue, respectively).

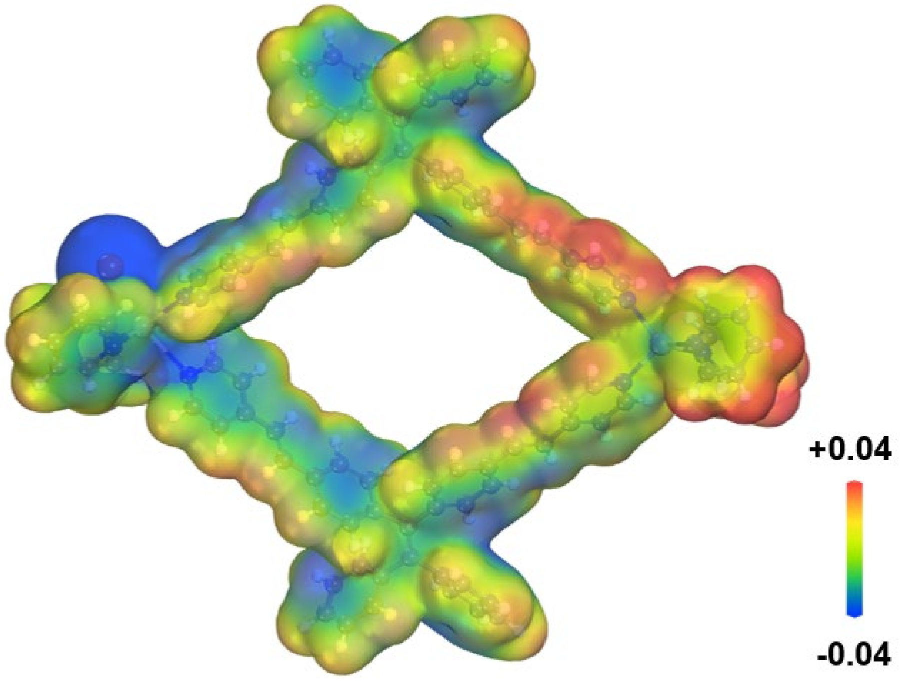

Figure S32. ESP mapping of the adsorption configuration of MOF-monoAg after binding to I^- ion.

Table S1. Crystal data and structure refinement for MOF-monoAg.

	MOF-monoAg
Empirical formula	$C_{54}H_{40}AgBF_4N_4$
Formula weight	939.58
CCDC	2389331
Temperature/K	193.00
Crystal system	orthorhombic
Space group	*P*ccn
a/Å	15.0724(9)
b/Å	18.3878(10)
c/Å	25.3142(15)
α/°	90
β/°	90
γ/°	90
Volume/Å^3	7015.8(7)
Z	4
ρ_{calc}g/cm^3	0.890
μ/mm^{-1}	0.325
F(000)	1920.0
Crystal size/mm^3	0.23 × 0.19 × 0.1
Radiation	MoKα (λ = 0.71073)
Index ranges	$-17 \leq h \leq 17$, $-21 \leq k \leq 21$, $-30 \leq l \leq 30$
Reflections collected	37921
Independent reflections	6153 [R_{int} = 0.1188, R_{sigma} = 0.0789]
Data/restraints/parameters	6153/115/307
Goodness-of-fit on F^2	1.180
Final R indexes [I>=2σ (I)]	R_1 = 0.1055, wR_2 = 0.1685
Final R indexes [all data]	R_1 = 0.1479, wR_2 = 0.1838
Largest diff. peak/hole / e Å^{-3}	0.82/-0.49

Table S2. The fitting results of adsorption kinetics for I^- ions adsorption on MOF-monoAg.

Pseudo-first-order			Pseudo-second-order		
q_e (mg g^{-1})	k_1 (g mg^{-1} min^{-1})	R^2	q_e (mg g^{-1})	k_2 (g mg^{-1} min^{-1})	R^2
153.4	0.32	0.8880	173.0	0.002	0.9823

Table S3. Elemental analysis of MOF-monoAg.

	C%	N%	Ag%
Theoretical content	69.02%	5.96%	11.38%
Experimental content	67.81%	5.88%	11.05%

Table S4. The fitting results of the adsorption isotherm fitted by the Langmuir and the Freundlich models for aqueous I^- ions adsorption on MOF-monoAg.

Langmuir			Freundlich		
q_{mL} (mg g^{-1})	K_L (L mg^{-1})	R^2	K_F ((mg g^{-1}) (L mg^{-1})$^{1/n}$)	$1/n_F$	R^2
175.5	0.13	0.9693	35.77	0.33	0.7275

Table S5. Comparison of the distribution coefficient K_d (mL g^{-1}) and the adsorption rates (mg g^{-1} min^{-1}) for I^{-} ions adsorption of currently available adsorbents.

Adsorbents	K_d (mL g^{-1})	Adsorption rate (mg g^{-1} min^{-1})	Reference
Ag@Cu–C	302	0.26	[8]
Ag-MSHC-6	2513	6.42	[9]
CCF/CN@Ag/AgCl	1317	7.22	[10]
Ag_2O–SNF	302	2.67	[11]
Ag^+@UiO-66-$(COOH)_2$	241	3.69	[12]
MIL-101(Cr)-SO_3Ag	118	0.38	[13]
Ag^0-UiO-66-$(OH)_2$	725	2.42	[14]
$Ag_{1/3}$@Zn_3Al_1- LDH	56	0.02	[15]
$Ag_{2/3}$@Zn_3Al_1- LDH	83	0.03	[15]
Ag_1@Zn_3Al_1- LDH	153	0.05	[15]
$Ag_{4/3}$@Zn_3Al_1- LDH	206	0.06	[15]
15%-Bi@MIL	1802	8.43	[16]
δ-Bi_2O_3@PES	236	0.16	[17]
D-CuO/Cu_2O	621	0.31	[18]
Cu_2O@Cu/Al-CLDH	332	0.52	[19]
COF-V	1061	0.49	[20]
N-AF	186	3.84	[21]
CoAl LDH	71	3.71	[22]
NiAl LDH	98	3.44	[22]
MgFe-4C-LDH	342	0.001	[23]
MOF-monoAg	11630	10.48	This study

Table S6. The concentrations of anions in simulated Hanford wastewater.[24]

Anion	Concentration (mol L^{-1})
I^{-}	1.57×10^{-4}
NO_3^{-}	6.07×10^{-2}
NO_2^{-}	1.69×10^{-1}
Cl^{-}	6.39×10^{-2}
SO_4^{2-}	6.64×10^{-6}

Table S7. Concentrations of Ag^+ and BF_4^- in seawater before and after iodine extraction from seawater.

	Ag^+ concentration (ppm)[a)]	BF_4^- concentration (ppm)[b)]
Seawater	N.D.[c)]	1.3563
Seawater after iodine extraction	N.D.	1.3617

[a)]Concentrations of Ag^+ determined by ICP-MS. [b)]Concentrations of BF_4^- determined by ion chromatography. [c)]N.D. means not detected. The limit of detection is 0.1 ppb for Ag^+.

Table S8. The concentrations of anions in iodine-exposed water.[25]

Anion	Concentration (ppm)
I^-	5
Cl^-	80
$NO3^-$	5
$H_2PO_4^-$	5
SO_4^{2-}	20

References

[1] J. VandeVondele, M. Krack, F. Mohamed, M. Parrinello, T. Chassaing, J. Hutter. Quickstep: Fast and accurate density functional calculations using a mixed Gaussian and plane waves approach. *Comput. Phys. Commun.* **2005**, *167*, 103-128.

[2] S. Goedecker, M. Teter, J. Hutter. Separable Dual-Space Gaussian Pseudopotentials. *Phys. Rev. B* **1996**, *54*, 1703-1710.

[3] C. Hartwigsen, S. Goedecker, J. Hutter. Relativistic Separable Dual-Space Gaussian Pseudopotentials from H to Rn. *Phys. Rev. B* **1998**, *58*, 3641-3662.

[4] M. Krack, M. Parrinello. All-electron ab-initio Molecular Dynamics. *Phys. Chem. Chem. Phys.* **2000**, *2*, 2105-2112.

[5] J. VandeVondele, J. Hutter. Gaussian Basis Sets for Accurate Calculations on Molecular Systems in Gas and Condensed Phases. *J. Chem. Phys.* **2007**, *127*, 114105.

[6] J. P. Perdew, K. Burke, M. Ernzerhof. Generalized gradient approximation made simple. *Phys. Rev. Lett.* **1996**, *77*, 3865.

[7] S. Grimme, J. Antony, S. Ehrlich, H. Krieg. A Consistent and Accurate ab initio Parametrization of Density Functional Dispersion Correction (DFT-D) for the 94 Elements H-Pu. *J. Chem. Phys.* **2010**, *132*, 154104.

[8] C. H. Gong, Z. Y. Li, K. W. Chen, A. T. Gu, P. Wang, Y. Yang. Synthesis and characterization of Ag@Cu-based MOFs as efficient adsorbents for iodine anions removal from aqueous solutions. *J. Environ. Radioact.* **2023**, *265*, 107211.

[9] H. L. Li, Y. Li, B. L. Li, D. B. Liu, Y. Z. Zhou. Highly selective anchoring silver nanoclusters on MOF/SOF heterostructured framework for efficient adsorption of radioactive iodine from aqueous solution. *Chemosphere* **2020**, *252*, 126448.

[10] B. S. Li, F. Mumtaz, X. Li, M. R. Al Shehhi, K. Wang. A chitosan film implanted with g-C_3N_4@Ag nanosheets and in-suit formed AgCl nanoparticles for efficient iodide removal. *Chem. Eng. J.* **2023**, *470*, 144369.

[11] W. J. Mu, Q. H. Yu, X. L. Li, H. Y. Wei, Y. Jian. Adsorption of radioactive iodine on surfactant-modified sodium niobate. *RSC Adv.* **2016**, *6*, 81719-81725.

[12] J. Zhang, S. L. Yang, L. Shao, Y. M. Ren, J. L. Jiang, H. S. Wang, H. Tang, H. Deng, T. F. Xia. Highly Sensitive Adsorption and Detection of Iodide in Aqueous Solution by a Post-Synthesized Zirconium-Organic Framework. *Molecules* **2022**, *27*, 8547.

[13] X. D. Zhao, X. Han, Z. J. Li, H. L. Huang, D. H. Liu, C. L. Zhong. Enhanced removal of iodide from water induced by a metal-incorporated porous metal-organic framework. *Appl. Surf. Sci.* **2015**, *351*, 760-764.

[14] T. Wang, H. F. Zhao, X. D. Zhao, D. H. Liu. One-step preparation of Ag^0-MOF composites for effective removal of iodide from water. *J. Solid State Chem.* **2022**, *305*, 122680.

[15] X. Yuan, W. Yu, X. Xiao, L. Wang, Q. Wan. Removal of iodide from aqueous solutions using a silver-modified ZnAl layered double hydroxide. *J. Solid State Chem.* **2024**, *335*, 124731.

[16] W. J. Xu, W. S. Zhang, J. X. Kang, B. J. Li. Facile synthesis of mesoporous Fe-based MOFs loading bismuth with high speed adsorption of iodide from solution. *J. Solid State Chem.* **2019**, *269*, 558-565.

[17] Q. Zhao, G. Y. Chen, Z. R. Wang, M. Jiang, J. R. L. Lin, L. Zhang, L. Zhu, T. Duan. Efficient removal and immobilization of radioactive iodide and iodate from aqueous solutions by bismuth-based composite beads. *Chem. Eng. J.* **2021**, *426*, 131629.

[18] P. Wang, C. H. Gong, A. Y. Tang, A. T. Gu, K. W. Chen, Y. Yi. Cu-BTC derived CuO and CuO/Cu_2O composite: an efficient adsorption material to iodide ions. *Mater. Res. Express* **2023**, *10*, 025005.

[19] C. H. Gong, Z. Y. Li, K. W. Chen, A. T. Gu, P. Wang, Y. Yang. Synthesis and characterization of Cu_2O@Cu/Al-CLDH for efficient adsorption of iodide anions in aqueous solutions. *J. Radioanal. Nucl. Chem.* **2023**, *332*, 2793-2805.

[20] X. H. Tian, G. W. Zhou, J. W. Xi, R. F. Sun, X. F. Zhang, G. Wang, L. Mei, C. Hou, L. P. Jiang, J. H. Qiu. Vinyl-functionalized covalent organic frameworks for effective radioactive iodine capture in aqueous solution. *Sep. Purif. Technol.* **2023**, *310*, 123160.

[21] Z. Liao, N. Pan, J. Liu, C. Ma, X. Xia, J. Deng, G. Yang, X. Li, Z. Chen, W. Cheng, W. Zhang, X. Nie, F. Dong. Highly efficient iodide adsorption from medical radioactive wastewater by strong alkaline anion exchange fiber. *J. Environ. Chem. Eng.* **2024**, *12*, 111783.

[22] J. Kang, F. Cintron-Colon, H. Kim, J. Kim, T. Varga, Y. G. Du, O. Qafoku, W. Um, T. G. Levitskaia. Removal of iodine (I^- and IO_3^-) from aqueous solutions using CoAl and NiAl layered double hydroxides. *Chem. Eng. J.* **2022**, *430*, 132788.

[23] J. Kang, T. G. Levitskaia, S. Park, J. Kim, T. Varga, W. Um. Nanostructured MgFe and CoCr layered double hydroxides for removal and sequestration of iodine anions. *Chem. Eng. J.* **2020**, *380*, 122408.

[24] L. Zhu, D. P. Sheng, C. Xu, X. Dai, M. A. Silver, J. Li, P. Li, Y. X. Wang, Y. L. Wang, L. H. Chen, C. L. Xiao, J. Chen, R. H. Zhou, C. Zhang, O. K. Farha, Z. F. Chai, T. E. Albrecht-Schmitt, S. Wang. Identifying the Recognition Site for Selective Trapping of $^{99}TcO_4^-$ in a Hydrolytically Stable and Radiation Resistant Cationic Metal-Organic Framework. *J. Am. Chem. Soc.* **2017**, *139*, 14873-14876.

[25] M. Zhang, J. Samanta, B. A. Atterberry, R. Staples, A. J. Rossini, C. Ke. A Crosslinked Ionic Organic Framework for Efficient Iodine and Iodide Remediation in Water. *Angew. Chem. Int. Ed.* **2022**, *61*, e202214189.
